# Culture, Sex, and Group-Bias in Trait and State Empathy

**DOI:** 10.3389/fpsyg.2021.561930

**Published:** 2021-04-28

**Authors:** Qing Zhao, David L. Neumann, Chao Yan, Sandra Djekic, David H. K. Shum

**Affiliations:** ^1^Department of Pain Management, The State Key Clinical Specialty in Pain Medicine, The Second Affiliated Hospital of Guangzhou Medical University, Guangzhou, China; ^2^School of Applied Psychology, Griffith University, Brisbane, QLD, Australia; ^3^Menzies Health Institute Queensland, Griffith University, Brisbane, QLD, Australia; ^4^School of Applied Psychology, Griffith University, Gold Coast, QLD, Australia; ^5^Key Laboratory of Brain Functional Genomics (MOE&STCSM), Shanghai Changning-ECNU Mental Health Center, School of Psychology and Cognitive Science, East China Normal University, Shanghai, China; ^6^School of Applied Arts, University of Art in Belgrade, Belgrade, Serbia; ^7^Department of Rehabilitation Sciences, The Hong Kong Polytechnic University, Kowloon, Hong Kong; ^8^Neuropsychology and Applied Cognitive Neuroscience Laboratory, Key Laboratory of Mental Health, Institute of Psychology, Chinese Academy of Sciences, Beijing, China

**Keywords:** culture–sex interaction, ethnic group bias, sex group favor, Empathy Quotient (EQ), Interpersonal Reactivity Index (IRI)

## Abstract

Empathy is sharing and understanding others’ emotions. Recently, researchers identified a culture–sex interaction effect in empathy. This phenomenon has been largely ignored by previous researchers. In this study, the culture–sex interaction effect was explored with a cohort of 129 participants (61 Australian Caucasians and 68 Chinese Hans) using both self-report questionnaires (i.e., Empathy Quotient and Interpersonal Reactivity Index) and computer-based empathy tasks. In line with the previous findings, the culture–sex interaction effect was observed for both trait empathy (i.e., the generalized characteristics of empathy, as examined by the self-report questionnaires) and state empathy (i.e., the on-spot reaction of empathy for a specific stimulus, as evaluated by the computer-based tasks). Moreover, in terms of state empathy, the culture–sex interaction effect further interacted with stimulus traits (i.e., stimulus ethnicity, stimulus sex, or stimulus emotion) and resulted in three- and four-way interactions. Follow-up analyses of these higher-order interactions suggested that the phenomena of ethnic group bias and sex group favor in empathy varied among the four culture–sex participant groups (i.e., Australian female, Australian male, Chinese female, and Chinese male). The current findings highlighted the dynamic nature of empathy (i.e., its sensitivity toward both participant traits and stimulus features). Furthermore, the newly identified interaction effects in empathy deserve more investigation and need to be verified with other Western and Asian populations.

## Introduction

*Empathy* is the sharing and understanding of others’ emotions (Cohen and Strayer, 1996; Eslinger, 1998; Zhao et al., 2019) and is an essential social skill (Baron-Cohen and Wheelwright, 2004). This skill can be evaluated in terms of trait and state empathy (Van der Graaff et al., 2016). *Trait empathy* reflects one’s general characteristics of empathy (Song et al., 2019), commonly gauged by self-report questionnaires (e.g., Zhao et al., 2019). *State empathy* represents one’s context-specific empathy toward a given stimulus (Song et al., 2019), usually evaluated through computer-based tasks (e.g., Neumann et al., 2013). Recently, researchers identified a significant culture–sex interaction effect on trait empathy, suggesting a combined impact of cultural background and biological sex on one’s empathy (Zhao et al., 2019). However, to date, the interaction effect has only been explored in terms of the trait (e.g., Melchers et al., 2015; Zhao et al., 2019) but not state empathy. The current study aims to examine the culture–sex interaction effect on both trait and state empathy with a cohort of Australian and Mainland Chinese participants.

In the research field of the cross-cultural comparison of empathy, *culture* is defined as a multi-faceted concept, covering a plethora of factors, including the local customs, country of origin, ethnicity, and native language (Zhao et al., 2019). Although culture is deemed to have an important impact on empathy, the current conclusions regarding the cultural impact on trait and state empathy are inconsistent among publications (see Tables 1–3). This inconsistency may be decoded from three aspects. First, empathy consists of two main components: emotional empathy and cognitive empathy (Cohen and Strayer, 1996; Shamay-Tsoory et al., 2009; Shamay-Tsoory, 2011). These two components work in unison to process others’ emotions but with different emphases. *Emotional empathy* is the more rudimentary form of empathy, representing the automatic sharing of others’ feelings (Shamay-Tsoory et al., 2009). *Cognitive empathy* is the more advanced form of empathy, representing the understanding of others’ emotions (Shamay-Tsoory et al., 2009). As summarized in Tables 1, 2, the cultural impact on each evaluation of empathy (e.g., emotional, cognitive, and overall empathy) could be inconsistent.

**TABLE 1:** A literature review of Western-Asian cross-cultural comparisons of trait empathy using self-report scales.

		Western culture		Asian culture		Western-Asian comparisons^†^		
References	Empathy scales	Participants (*n*)	Male%	Participants (*n*)	Male%	EQ-40	IRI-PT	IRI-EC
Xu et al., 2009	IRI	Caucasian^a^ (16)	50	Mainland Chinese^b^ (17)	47	/	W > A	W > A
Cassels et al., 2010	IRI	Western^a^ (32)	/	Asian^a^ (74)	/	/	/	W > A
de Greck et al., 2012	IRI	German Caucasian (16)	38	Mainland Chinese Han (16)	38	/	W = A	W > A
Kaelber and Schwartz, 2014	IRI	American^c^ (53)	19	Thai^c^ (48)	31	/	W > A	W > A
Jiang et al., 2014	IRI	Western^a^ (18)	0	Mainland Chinese^b^ (18)	0	/	W = A	W = A
Melchers et al., 2015	EQ, IRI	German^b^ (202)	25	Mainland Chinese^b^ (438)	62	W > A	W = A	W = A
Melchers et al., 2016	EQ, IRI	German^b^ (304)	24	Mainland Chinese^b^ (438)	62	W > A	W = A	W = A
		Spanish^b^ (62)	44	Mainland Chinese^b^ (438)	62	W = A	W = A	W = A
		American^b^ (92)	39	Mainland Chinese^b^ (438)	62	W = A	W = A	W = A
Lachmann et al., 2018	IRI	German^b^ F (207)	0	Mainland Chinese^b^ F (162)	0	/	W > A	W > A
		German^b^ M (97)	100	Mainland Chinese^b^ M (450)	100	/	W > A	W = A
Zhao et al., 2019	EQ, IRI	Australian Caucasian F (95)	0	Mainland Chinese Han F (152)	0	W > A	W > A	W > A
		Australian Caucasian M (101)	100	Mainland Chinese Han M (59)	100	W = A	W = A	W = A
The current	EQ, IRI	Australian Caucasian F (32)	0	Mainland Chinese Han F (36)	0	W > A	W > A	W > A
		Australian Caucasian M (29)	100	Mainland Chinese Han M (32)	100	W = A	W = A	W = A

*EQ, Empathy Quotient; EQ-40, total score for the 40-item EQ; IRI, Interpersonal Reactivity Index; IRI-PT, total score for the IRI perspective-taking items; IRI-EC, total score for the IRI empathic concern items. ^†^For the Western-Asian comparisons: W, the Western participants; A, the Asian participants. ^abc^For the participant, ^a^multi-nationalities; ^b^the ethnicity was unknown; ^c^multi-ethnicities. F, female participants; M, male participants.*

**TABLE 2:** A literature review of Western-Asian cross-cultural comparisons of state empathy using computer-based tasks.

	Stimulus and task information				Western culture		Asian culture		Western-Asian comparisons^†^		
References	Stimulus type^■^	Stimulus ethnicity	Stimulus emotion	Empathy task^∗^	Participants (*n*)	Male%	Participants (*n*)	Male%	Cultural difference	Ethnic in-group bias	Ethnic out-group bias
Cheon et al., 2011	Picture	Combined	Suffering	OE	American Caucasian (14)	50	Korean (13)	62	/	× W ✓ A	× W × A
	Picture	Combined	Neutral	OE	American Caucasian (14)	50	Korean (13)	62	/	× W × A	× W × A
Neumann et al., 2013	Picture	Combined	Positive	EE	Caucasian^a^ (99)	26	Mainland Chinese^b^ (99)	29	/	× W × A	× W ✓ A
				CE	Caucasian^a^ (99)	26	Mainland Chinese^b^ (99)	29	/	✓ W × A	× W × A
				PT	Caucasian^a^ (99)	26	Mainland Chinese^b^ (99)	29	/	× W × A	× W × A
	Picture	Combined	Negative	EE	Caucasian^a^ (99)	26	Mainland Chinese^b^ (99)	29	/	✓ W ✓ A	× W × A
				CE	Caucasian^a^ (99)	26	Mainland Chinese^b^ (99)	29	/	✓ W ✓ A	× W × A
				PT	Caucasian^a^ (99)	26	Mainland Chinese^b^ (99)	29	/	✓ W ✓ A	× W × A
Atkins et al., 2016	Video	Caucasian	Suffering	EE	British Caucasian (47)	17	Hong Kong Chinese (47)	28	W > A	× W × A	× W × A
	Video	Asian	Suffering	EE	British Caucasian (47)	17	Hong Kong Chinese (47)	28	W > A		
	Video	Caucasian	Suffering	CE	British Caucasian (47)	17	Hong Kong Chinese (47)	28	W = A	× W × A	× W × A
	Video	Asian	Suffering	CE	British Caucasian (47)	17	Hong Kong Chinese (47)	28	W < A		

^■^For the stimulus type, Atkins et al. (2016) used video clips in which the main character orally related their negative social experience; in contrast, the other researchers used static pictures with a relevant emotional background (e.g., a natural disaster or a social activity). *For the empathy task, OE, the overall empathy (i.e., the integrated concept of emotional and cognitive empathy); EE, the emotional empathy; CE, the cognitive empathy; PT, the perspective-taking. ^†^For the Western-Asian comparisons, W, Western participants; A, Asian participants; ✓, showed; ×, did not show. ^ab^For the participant, ^a^multi-nationalities; ^b^multi-ethnicities.

**TABLE 3:** A literature review of Western-Asian cross-cultural comparisons of state empathy using fMRI.

	Stimulus and task information				Western culture		Asian culture		Western–Asian comparisons^†^	
References	Stimulus type^■^	Stimulus emotion	fMRI contrast	Empathy task*	Participants (*n)*	Male%	Participants (*n*)	Male%	W > A	W < A
Cheon et al., 2011	Picture	Suffering	In-group vs. Out-group	OE	American Caucasian (14)	50	Korean (13)	62	rBA19	bBA40; mBA24; lBA19/37
de Greck et al., 2012	Picture	Angry	In-group vs. Baseline	OE	German Caucasian (16)	38	Mainland Chinese Han (16)	38	rBA20/22/40; lBA13	lBA9
		Neutral	In-group vs. Baseline	OE	German Caucasian (16)	38	Mainland Chinese Han (16)	38	rBA20; lBA13	lBA9

^■^For the stimulus type, Cheon et al. (2011) presented emotional pictures with a relevant emotional background (e.g., a natural disaster or a social activity), while de Greck et al. (2012) presented photos of facial expressions with a plain back-drop. *For the empathy task, OE, the overall empathy (i.e., the integrated concept of emotional and cognitive empathy). ^†^For the Western–Asian comparisons, W, Western participants; A, Asian participants; b/l/m/rBA, the bilateral, left, middle, and right side/s of a Brodmann Area, correspondingly.

Second, among the previous publications, researchers have primarily made comparisons of empathy between Western and Asian cultures (Tables 1–3). These two cultures present a sharp contrast of self-construal (Singelis, 1994; Zhao et al., 2019). *Self-construal* is the psychological distance between the self and others and is the pillar of an individual’s psychological reactions (Singelis, 1994). For example, Zhao et al. (2019) identified that Australian and Chinese participants represented independent and interdependent self-construal, respectively. Moreover, with the Australian and Chinese participants, Zhao et al. (2019) found that self-construal was a mediator of the prediction from culture to trait empathy; however, the mediating effect varied between female and male participant groups^1^, indicating the culture–sex interaction effect in empathy in a broader sense (also see Schmitt, 2015). Nevertheless, the complicated issue is that the definitions of the Western and Asian cultures varied among previous studies (Tables 1–3). Some researchers referred to “culture” as the cultural background (e.g., Australians and Chinese locals recruited from their home country; Zhao et al., 2019), some researchers referred to it as ethnicity (e.g., Caucasian and Chinese participants; Xu et al., 2009), and some others referred to it as country of origin (e.g., Western and Asian born students, studying in Canada; Cassels et al., 2010). Furthermore, some researchers recruited participants with mixed cultural backgrounds (e.g., Cassels et al., 2010), mixed ethnicities (e.g., Kaelber and Schwartz, 2014), or mixed countries of origin (e.g., Xu et al., 2009).

Third, the impact of sex has been largely ignored by previous researchers (Tables 1–3). However, Zhao et al. (2019) proposed that one’s cultural background and biological sex might interact to determine the participant’s empathy. Specifically, they found that cultural differences in trait empathy (i.e., Australian > Chinese participant) were only significant with the female but not male participants (Zhao et al., 2019). Meanwhile, they found that the sex difference (i.e., female > male participant) in trait empathy was only significant for the Australian but not Chinese participants (Zhao et al., 2019). Moreover, they reported that the above culture–sex interaction effect was significant on all forms of trait empathy (i.e., emotional, cognitive, and overall empathy) (Zhao et al., 2019). Among the previous researchers (Tables 1–3), only Melchers et al. (2015) examined the culture–sex interaction effect in trait empathy. Interestingly, they identified a similar^2^ effect with German and Chinese participants (Melchers et al., 2015). In contrast, most of the other researchers examined the cultural impact on empathy *per se* but neglected to evaluate the possible culture–sex interaction effect. Based on the findings by Zhao et al. (2019), the sex ratio of participant samples could confound the results of cultural differences in empathy. In other words, among the previous studies (Tables 1–3), the larger Western–Asian cultural difference in empathy was likely to be reported by these studies with more female participants (i.e., larger female%) (see a discussion by Zhao et al., 2019).

The culture–sex interaction effect in empathy could be due to the inconsistent social expectations for sex roles across cultures (Zhao et al., 2019). In Western cultures, females are expected to be warm-hearted and openly affectionate, while males are encouraged to be strong-willed and emotionally invulnerable (Jaggar, 1989; Merten, 2005). In contrast, the above social expectation for sex differentiation could be smaller in Asian than in Western cultures; for example, according to the traditional Chinese culture, both Chinese males and Chinese females are expected to seek a balance between *Yin* (e.g., negative and femininity) and *Yang* (e.g., positive and masculinity) (Huang, 2006; Zhao et al., 2019). Consistently, researchers found that the effect size of the sex difference on trait empathy is smaller for Asians (see a review by Zhao et al., 2019) than Westerners (see a review by Groen et al., 2015).

To date, the culture–sex interaction effect has been verified in trait (Melchers et al., 2015; Zhao et al., 2019) but not state empathy. This may reflect that the culture–sex interaction effect on state empathy is harder to detect relative to trait empathy. On the one hand, social expectations could have less impact on computer-based assessments of state empathy relative to self-report evaluations of trait empathy (Baez et al., 2017). If the culture–sex interaction effect in trait empathy is largely due to social expectations (Zhao et al., 2019), the interaction effect on state empathy will be smaller in magnitude. On the other hand, state empathy is sensitive not only to participant traits (i.e., participant culture and participant sex) but also to stimulus features (e.g., stimulus ethnicity, stimulus sex, and stimulus emotion). Consequently, the culture–sex interaction effect on state empathy could be statistically swamped by other phenomena of empathy (e.g., ethnic group biases and sex group favors), or even combined with the other effects to formulate higher-order interactions (e.g., three- to four-way interactions).

*Ethnic group bias* refers to the phenomenon that people have different inclinations in empathy toward individuals with the same or different ethnicities (Neumann et al., 2013). Commonly, there is an *ethnic in-group bias* in empathy (see Table 2); that is, people tend to show more empathy for the ethnic in-group individuals (i.e., those with the same ethnicity) than out-group ones (i.e., those with a different ethnicity) (Cheon et al., 2011; Neumann et al., 2013). However, an *ethnic out-group bias* in empathy (i.e., the converse trend as introduced) can also be observed (e.g., Neumann et al., 2013). According to findings reported by Neumann et al. (2013), Chinese participants showed an ethnic out-group bias in state empathy for positive emotions (e.g., happiness) and an ethnic in-group bias for negative emotions (e.g., sadness). In contrast, Australian participants had ethnic in-group biases for both positive and negative emotions (Neumann et al., 2013). The results of Neumann et al. (2013) revealed a three-way interaction between participant culture, stimulus ethnicity, and stimulus emotion. To date, higher-order interaction effects in empathy (e.g., three- or four-way interaction) that extend beyond the fundamental culture–sex interaction effect have not been examined (see Tables 1–3).

The origin of the ethnic group bias in empathy is intricate, and three theories have been proposed. The first is “in-group familiarity” (Cao et al., 2015). Researchers have found that the mirror neuron system (i.e., a key brain region for empathy) (Shamay-Tsoory et al., 2009) tends to be activated more by familiar than unfamiliar stimuli (Platek et al., 2006). Meanwhile, Cao et al. (2015) observed that increasing the out-group contact (i.e., reducing the ethnic out-group unfamiliarity) might help participants to reduce ethnic in-group bias in empathy. Specifically, they found that Chinese overseas students with greater daily-life contact with Caucasians reacted to Caucasian characters in pain with higher brain activation in the anterior cingulate cortex (i.e., a brain area related to empathy for pain) (Cao et al., 2015).

The second theory is “out-group hate” (Avenanti et al., 2010). Avenanti et al. (2010) presented American participants with pictures of three ethnic hands being pricked by needles. They found that both Caucasian and African American participants exhibited strong pain-related empathic reactivity for the hand of their own ethnicity but not for the hand of the other ethnicity (Avenanti et al., 2010). Intriguingly, both groups of participants showed moderate empathic reactivity for the hand that belonged to the most unfamiliar and prejudice-free “ethnicity” (i.e., it is an artificial ethnicity with its skin pigmented into violet) (Avenanti et al., 2010). It should be noted that although the theories of both in-group familiarity (Cao et al., 2015) and out-group hate (Avenanti et al., 2010) could explain the ethnic in-group bias in empathy, neither could explain the ethnic out-group bias in empathy (e.g., Neumann et al., 2013).

Instead, both ethnic in-group and out-group biases in empathy might be explained by a third theory called “reciprocal altruism” (Trivers, 1971; Mathur et al., 2010). According to Mathur et al. (2010), for preserving resources within a reciprocal group, people tend to express altruism toward reciprocal individuals (e.g., ethnic in-group) over non-reciprocal ones (e.g., ethnic out-group). It should be emphasized that empathy is an essential motivator of altruism (Mathur et al., 2010). Meanwhile, either people showing in-group or out-group bias in empathy might be tipped by the valence of emotions (Neumann et al., 2013). First, negative emotions (e.g., sadness, fear, and anger) reveal one’s powerlessness (Merten, 2005) or dissatisfaction (Wubben et al., 2011), which may elicit help and cooperative behaviors from others (Hackenbracht and Tamir, 2010; Wubben et al., 2011). Second, positive emotions (e.g., happiness) commonly represent feelings of joyfulness and contentment; however, a smile can also be applied to conceal negative inner thoughts (e.g., sadness and anger) (Ekman et al., 1988; Svetieva et al., 2019). Moreover, Tan and Forgas (2010) found that participants in a happy mood, relative to those in a sad mood, were greedier during resource distribution among themselves and others. It should be noted that if an ethnic out-group person in a happy mood tends to be greedy, the resource distribution will be non-reciprocal. Hence, it may be the case that showing more concerns for in-group suffering (i.e., a proclivity to share resources reciprocally) and being more concerned by out-group happiness (i.e., a sensitivity to a potential resource loss) complementarily reflect the theory of reciprocal altruism (Mathur et al., 2010).

The current study aimed to explore the culture–sex interaction effect in both trait and state empathy with Australian and Chinese participants. Trait empathy was evaluated by two self-report questionnaires: the Empathy Quotient (EQ; Baron-Cohen and Wheelwright, 2004) and Interpersonal Reactivity Index (IRI; Davis, 1980). State empathy was assessed by computer-based tasks adapted from Neumann et al. (2013). Referencing Zhao et al. (2019), the effect of culture–sex interaction in empathy was expected to be verified in both trait and state empathy. Referencing Neumann et al. (2013), both ethnic in- and out-group biases in empathy were anticipated, which might further interact with the culture–sex interaction effect on state empathy. Finally, apart from the ethnic group biases, we considered that participants might adjust their empathy according to stimulus sex (i.e., the sex group favor). Since the sex group favor effect in empathy was not examined in previous studies (Tables 1–3), no specific hypotheses were made on this point.

## Materials and Methods

### Participants

Participants in this study (i.e., Australian Caucasians and Chinese Hans) were recruited from their home countries (i.e., Australia and China, correspondingly). Both Caucasian and Han are the main ethnicity of the respective countries. In this study, *Participant Culture* is defined as an integrated concept, covering the mainstream culture (i.e., the Western culture and the Chinese culture), ethnicity (i.e., Caucasian and Chinese Han), country of origin (i.e., Australia and China), as well as the official language (i.e., English and Chinese). In China, the official spoken language is Mandarin, and the official written language is the simplified Chinese characters. Both the official spoken and written Chinese were used in the current study.

The inclusion criteria of the current study were presented for participants at the beginning of each experiment (criteria for Australians/Chinese, respectively): (1) nationality was Australian/Chinese; (2) ethnicity was Caucasian/Han; (3) place of birth was Australia/Chinese Mainland; (4) the main place of growing up was Australia/Chinese Mainland; (5) the main place of residence was Australia/Chinese Mainland (6) 18 years or older; (7) first- or second-year undergraduate student; (8) normal or corrected normal vision; and (9) without any history of mental or neurological illness. Moreover, it was explained to all participants that the current investigation was restricted to the ones satisfying all of the above inclusion criteria.

An English–Mandarin bilingual researcher administered the current investigation. Both Australian and Chinese participants received instructions and completed the tasks in their native languages. Participants were required to provide informed consent online at the beginning of each investigation. After completing the tasks, each participant was reimbursed according to the ethical protocols (i.e., US$6 for a Chinese participant, US$11 for an Australian participant, or two study credits for a participant of either cultural group). The ethical protocols for conducting the current study were provided by an ethics committee in both Australia and China.

In total, 139 people were recruited^3^. Ten of them were excluded for the following reasons: one withdrew from the experiment; one did not finish the task due to a technical issue; two were identified as not taking the tasks seriously (e.g., repeatedly selecting the same option for items of trait empathy); and six did not satisfy the inclusion criteria (identified afterward according to the information they provided on the online survey, such as having a history of coma or depression). Therefore, the final sample size was 129, with 61 Australian Caucasian university students (32 females, mean age = 19.37 years, *SD* = 1.39; 29 males, mean age = 19.69 years, *SD* = 1.67) and 68 Chinese Han university students (36 females, mean age = 19.47 years, *SD* = 0.97; 32 males, mean age = 19.69 years, *SD* = 0.69).

### Self-Report Questionnaires

#### Demographic Sheet

Participants’ responses for self-report questionnaires were collected online^4^. On the demographic sheet, participants’ age, sex, and other traits covered by the inclusion criteria were enquired about.

#### Empathy Quotient

Empathy Quotient is a self-report questionnaire that measures trait empathy as a single concept (i.e., an evaluation of the overall trait empathy) (Baron-Cohen and Wheelwright, 2004). EQ consists of 40 empathy items and 20 filler items; according to the official rule, these filler items are not counted for the total score of the EQ (Baron-Cohen and Wheelwright, 2004). These filler items were designed by the original authors to keep participants from continually answering empathy-related questions (Baron-Cohen and Wheelwright, 2004). The empathy items should be scored according to the official rule and summed up to a total score ranging from 0 to 80, with a higher score reflecting a higher overall trait empathy (Baron-Cohen and Wheelwright, 2004). The original English version (Baron-Cohen and Wheelwright, 2004) and the simplified Chinese-translated version (Zhao et al., 2018) of the EQ were administered in this study for Australian and Chinese participants, respectively. Based on the current participants, Cronbach’s αs for the English and the simplified Chinese-translated versions of the EQ were 0.92 and 0.90, correspondingly. The current results of the internal consistency were similar to previous reports for the EQ based on both Western (e.g., Groen et al., 2015) and Asian populations (e.g., Zhao et al., 2018).

#### Interpersonal Reactivity Index

Interpersonal Reactivity Index is a self-report questionnaire that assesses four aspects of trait empathy (Davis, 1980), namely, empathic concern (i.e., IRI-EC, seven items), perspective-taking (i.e., IRI-PT, seven items), personal distress (i.e., IRI-PD, seven items), and fantasy (i.e., IRI-FS, seven items). According to Davis (1980), IRI-EC and IRI-PT tap into emotional and cognitive trait empathy, respectively. IRI-PD evaluates one’s empathic personal distress (i.e., the aversive feelings when witnessing others’ suffering), while IRI-FS assesses one’s empathy for imaginary characters portrayed in photos, books, or movies (Davis, 1980). The total score of each subscale of IRI ranges from 0 to 28, with a higher score reflecting a stronger trait in the corresponding category. The original English (Davis, 1980) and the simplified Chinese-translated version of the IRI (Chan, 1986; Zhao et al., 2018) were administered in this study for Australian and Chinese participants, respectively. Cronbach’s αs of the four IRI subscales (viz., IRI-EC, IRI-PT, IRI-PD, and IRI-FS) of the English version were 0.84, 0.79, 0.64, and 0.83, in sequence, and of the simplified Chinese version were 0.75, 0.61, 0.73, and 0.86, correspondingly. These values of Cronbach’s αs were similar to previous findings based on both Western (e.g., Davis, 1980) and Asian participants (e.g., Zhao et al., 2019).

### Computer-Based Tasks

#### Instruction

Two computer-based tasks (i.e., tasks I and II) were administered in this study. The paradigms of both tasks were adapted from Neumann et al. (2013). Participants were asked to look at two cohorts of emotional stimuli and answer a set of empathy-related questions for each stimulus. Meanwhile, participants were instructed to be concerned with the feelings of the main character in the given stimuli (i.e., to test participants’ explicit empathy). Additionally, participants were told to respond to each question according to their own feelings, rather than focus on judging whether an answer was right or wrong (i.e., to minimize the impact of social expectations).

#### Parameters and Equipment

For each trial, a cross was presented as the central fixation (onscreen for 3,000 ms). Afterward, a stimulus was given (onscreen for 6,000 ms), followed by a series of task questions presented one by one (onscreen until the participants provided a valid answer). Stimuli were presented in a pseudo-random pattern and task questions were presented in a fixed pattern. E-prime 2.0 (Schneider et al., 2002) was used to present the tasks and collect participants’ responses. A 14-in. (35.56 cm) laptop was used in this study with both Australian and Chinese participants.

#### Stimuli

##### Stimuli of task I

In total, 24 stimuli of single-character portrait photos (2 stimulus ethnicity × 2 stimulus sex × 6 stimulus emotion) were extracted from the NimStim database^5^ (Tottenham et al., 2009). In terms of stimulus ethnicity, there were two ethnic groups (i.e., typical Caucasian and typical East Asian characters) presented with an equal presenting ratio (i.e., 50% Caucasian and 50% Asian characters). In terms of stimulus sex, female and male stimuli were given with an equal presenting ratio (i.e., 50% female and 50% male stimuli). In terms of stimulus emotion, six emotional types (i.e., happiness, anger, sadness, surprise, fear, and neutral-peacefulness) were presented with an equal presenting ratio (i.e., 16.7% per emotion). It should be noted that empathy for most emotions (i.e., happiness, anger, sadness, surprise, and fear) was straightforward. Specifically, in light of the neutral-peacefulness stimuli, participants were instructed to empathize with the peacefulness of the main character.

##### Stimuli of task II

In sum, 24 documentary photos (2 stimulus ethnicity × 2 stimulus sex × 6 stimulus emotion) were presented in task II. The stimulus sets of tasks I and II differed in two essential aspects. First, the stimuli of documentary photos (i.e., task II) captured the naturally expressed emotions; in contrast, the facial expressions of the NimStim models (i.e., task I) were posed under the instruction of Tottenham et al. (2009). Second, the stimuli of task II informed participants of the relevant emotional background (e.g., a wedding party or a work strike; see more details of the background information in Supplementary Document 1)^6^; however, the background of NimStim stimuli was a plain back-drop (Tottenham et al., 2009).

Since no published picture databases can provide the full stimulus set required by task II of this study, three resources were used. Four pictures^7^ were selected from the International Affective Picture System (IAPS; Lang et al., 1999), two pictures of happiness were obtained from Neumann et al. (2013), and the remaining were sourced from the Internet^8^. The final stimuli set of task II consisted of 10 single-character pictures and 14 multi-characters pictures. To help participants identify the main character, an arrow pointing to the main character was added to all task II stimuli. Except for the aforementioned aspects, the stimuli selection of task II mirrored the procedure of task I.

#### Task Questions

##### Validation questions

Four validation questions were presented after each stimulus. In sequence, the questions were (Q1) “How strongly was the main character feeling the emotion? 1 = *not at all* to 9 = *very strongly*”; (Q2) “How negative or positive did the picture seem? 1 = *very negative* to 9 = *very positive*”; (Q3) “Viewing the picture, I felt _____. 1 = *very calm/relaxed* to 9 = *very aroused/jittery*”; (Q4) “Viewing the picture, I felt _____. 1 = *very at ease/comfortable* to 9 = *very distressed*.” These four questions were used to record participants’ judgments on emotional intensity, emotional valence, emotional arousal, and emotional distress, respectively.

##### State empathy questions

Four questions were used to tap into state empathy, and they were (Q5) “What was the main emotion that the main character was feeling? 1 = *Happiness*, 2 = *Surprise*, 3 = *Neutral*, 4 = *Fear*, 5 = *Anger*, and 6 = *Sadness*”; (Q6) “I felt _____ the feeling of the main character. 1 = *not at all* to 9 = *very strongly*”; (Q7) “I understood _____ the situation of the main character. 1 = *not at all* to 9 = *very fully*”; and (Q8) “I can _____ imagine myself in the situation of the main character. 1 = *not at all* to 9 = *very easily*.” These four questions assessed participants’ empathic accuracy, emotional empathy, cognitive empathy, and perspective-taking, correspondingly. In light of task I, three questions of state empathy (i.e., Q5 to Q7) were asked, but without Q8, since the NimStim stimuli did not give a valid emotional background for participants to commit the perspective-taking. In light of task II, all four questions (i.e., Q5 to Q8) were administered.

#### Pilot Testing

Pilot testing (*n* = 7) was conducted to optimize and finalize the study procedure (see Supplementary Document 2).

### Data Analysis

#### Trait Empathy

A group of 2 (participant culture) × 2 (participant sex) ANOVAs (sum of squares type II) was conducted to verify the impacts of participant culture, participant sex, and the culture–sex interaction effect on trait empathy scores (i.e., the EQ and IRI scores). Furthermore, pairwise comparisons with Bonferroni adjustment (accounting for the inflated type I error) were carried out to identify the source of any significant interactions suggested by the ANOVAs.

#### Empathic Accuracies for Tasks I and II

An omnibus test of 2 (participant culture) × 2 (participant sex) × 2 (stimulus ethnicity) × 2 (stimulus sex) × 6 (stimulus emotion) generalized linear mixed models (GLMM) (Logistic regression) was conducted based on participants’ empathic accuracy (i.e., emotion recognition ACC; dummy coded, *0* = *inaccurate* and *1* = *accurate*) of stimuli tasks I and II, separately^9^. In this study, each participant had a corresponding ACC for each stimulus (i.e., in total 48 variables of ACC per participant). Therefore, the current authors firstly converted the data from the original wide version into a long version, and then conducted the above omnibus test based on the long version data. Moreover, since the empathic accuracy (i.e., ACC) might have an impact on participants’ responses of empathy (e.g., emotional and cognitive empathy), the current authors conducted further analyses of state empathy based on the long version data with the variable of ACC controlled as a covariate. Accordingly, the degree of freedom increased for the current analyses of state empathy (based on the long version data) relative to the analyses of trait empathy (based on the wide version data).

#### State Empathy for Task I

With ACC controlled as a covariate, an omnibus test^10^ of 2 (empathy task) × 2 (participant culture) × 2 (participant sex) × 2 (stimulus ethnicity) × 2 (stimulus sex) × 6 (stimulus emotion) linear mixed model (sum of squares type III) was carried out based on all state empathy responses collected in task I. If the above omnibus test indicated any significant interaction effects, pairwise comparisons (with Bonferroni adjustment) were conducted to identify the source of the interactions. Moreover, if the omnibus test indicated any significant interaction effects relevant to the variable of the ‘empathy task’, follow-up analyses^10^ were carried out. For the follow-up analyses, 2 (participant culture) × 2 (participant sex) × 2 (stimulus ethnicity) × 2 (stimulus sex) × 6 (stimulus emotion) linear mixed model (sum of squares type III) were conducted for the two categories of the ‘empathy task’ of task I (viz., emotional and cognitive empathy), separately (adjusted significance level α = 0.050/2; ACC was controlled as a covariate). Finally, pairwise comparisons (with Bonferroni adjustment) were carried out to identify the source of any significant interaction effects identified in the follow-up analyses.

#### State Empathy for Task II

The overall analysis procedure was similar to that of task I, but with two modifications. First, the ‘empathy task’ of task II consisted of three categories (viz., emotional empathy, cognitive empathy, and perspective-taking). Accordingly, the omnibus test of task II was 3 (empathy task) × 2 (participant culture) × 2 (participant sex) × 2 (stimulus ethnicity) × 2 (stimulus sex) × 6 (stimulus emotion) linear mixed model (sum of squares type III). Second, the adjusted significance level of the three follow-up analyses of task II was α = 0.05/3. The correlation coefficients between trait empathy and state empathy are presented in Supplementary Document 3. All analyses were conducted using SPSS version 27 (IBM Corp).

## Results

### Stimulus Validity

A summary of the stimulus validity of the current stimuli (i.e., empathic accuracy, emotional intensity, emotional valence, emotional arousal, and emotional distress) is presented in Supplementary Document 1.

### Self-Report Questionnaires

Means and standard deviations for the self-report scores (i.e., EQ, IRI-PT, IRI-EC, IRI-PD, and IRI-FS) are presented in Table 4. Results of 2 (participant culture) × 2 (participant sex) ANOVAs revealed significant participant culture–sex interactions on three trait empathy scores, namely, the EQ [*F*(1,125) = 5.24, *p* = 0.024, η_*p*_^2^ = 0.04], the IRI-PT [*F*(1,125) = 5.02, *p* = 0.027, η_*p*_^2^ = 0.04], and IRI-EC [*F*(1,125) = 9.70, *p* = 0.002, η_*p*_^2^ = 0.07]. In addition, the main effect of participant culture was identified on the IRI-PD (i.e., Chinese > Australian participant; *F*(1,125) = 20.02, *p* < 0.001, η_*p*_^2^ = 0.14). In contrast, no significant effects were observed on the IRI-FS (all *p*s ≥ 0.081).

**TABLE 4:** The main effects and the culture–sex interaction effect on trait empathy with the four participant groups.

	Australian female (*n* = 32)		Australian male (*n* = 29)		Chinese female (*n* = 36)		Chinese male (*n* = 32)		ANOVA comparison								
									Culture			Sex			Interaction		
	*M*	*SD*	*M*	*SD*	*M*	*SD*	*M*	*SD*	*F*	*p*	η_*p*_^2^	*F*	*p*	η_*p*_^2^	*F*	*p*	η_*p*_^2^
EQ-40	53.84	12.87	41.69	12.08	42.89	13.04	40.62	10.65	8.47	0.004	0.06	10.37	0.002	0.08	5.24	0.024	0.04
IRI-PT	20.59	4.82	18.00	3.60	17.31	4.01	17.78	2.74	7.21	0.008	0.05	2.04	0.156	0.02	5.02	0.027	0.04
IRI-EC	23.06	4.83	18.93	4.39	18.28	4.46	18.91	3.50	11.02	0.001	0.08	4.53	0.035	0.03	9.70	0.002	0.07
IRI-PD	12.28	4.08	10.97	3.81	15.03	4.46	14.81	4.11	20.02	<0.001	0.14	1.02	0.315	0.01	0.57	0.453	<0.01
IRI-FS	20.62	5.51	17.48	5.21	20.22	4.60	20.25	5.12	1.49	0.225	0.01	2.68	0.104	0.02	3.10	0.081	0.02

*EQ, Empathy Quotient; EQ-40, total score for the 40-item EQ; IRI, Interpersonal Reactivity Index; IRI-PT, total score for the IRI perspective-taking items; IRI-EC, total score for the IRI empathic concern items; IRI-PD, total score for the IRI personal distress items; IRI-FS, total score for the IRI fantasy items.*

In light of the significant culture–sex interactions on the trait empathy scores (i.e., EQ, IRI-PT, and IRI-EC), pairwise comparisons revealed two main findings. First, the participant culture effect was only significant with female participants (i.e., Australian female > Chinese female, *p* < 0.001, *d* = 0.66, 95% CI = [5.08, 16.83], for the EQ; *p* = 0.001, *d* = 0.62, 95% CI = [1.42, 5.15], for the IRI-PT; and *p* < 0.001, *d* = 0.81, 95% CI = [2.70, 6.87], for the IRI-EC) but not with male participants (all *p*s ≥ 0.735). Second, the participant sex effect was found in Australian participants (i.e., Australian female > Australian male; *p* < 0.001, *d* = 0.69, 95% CI = [5.95, 18.36], for the EQ; *p* = 0.010, *d* = 0.47, 95% CI = [0.63, 4.56], for the IRI-PT; and *p* < 0.001, *d* = 0.67, 95% CI = [1.94, 6.33], for the IRI-EC) but not Chinese participants (all *p*s ≥ 0.447).

### Empathic Accuracy

Omnibus tests revealed neither main nor interaction effects on the ACC of either the NimStim stimuli (i.e., task I, all *p*s ≥ 0.884) or the documentary stimuli (i.e., task II, all *p*s ≥ 0.968). Nevertheless, the ACC was still controlled in the following analyses of state empathy as a covariate.

### State Empathy for the NimStim Stimuli

A full introduction of the state empathy results is presented in Table 5 and summarized in Supplementary Document 4. Due to the word limit of the main text, only significant interactions with participant traits, not being qualified by higher-order interactions, are presented in the following.

**TABLE 5:** The main and interaction effects on state empathy for the NimStim stimuli (task I) and the documentary stimuli (task II).

	Main effects						Interaction effects^■^		
	Empathy	Participant traits		Stimulus traits					
Analysis	Task^⋇^ (A)	Culture (B)	Sex^†^ (C)	Ethnicity^∵^ (D)	Sex^†^ (E)	Emotion♥ (F)	2-way	3-way	4-way
***The NimStim stimuli (task I)***									

**The omnibus test (α = 0.050)**	×	×	×	×	√***(f > m)	√***	√***(A × **B**); √***(A × **C**);	√*(**B** × D × F)	√*(**B** × **C** × D × E)
							√***(**B** × F); √**(**C** × F);		
							√***(D × F); √***(A × F).		
***Two follow-up tests (*α*’* = *0.025)***									
(1) Emotional empathy	/	×	×	×	√**(f > m)	√***	√***(**B** × F); √***(**C** × F);	×	×
							√**(D × F).		
(2) Cognitive empathy	/	×	×	×	√**(f > m)	√***	√***(**B** × F); √*(**B** × E);	×	√*(**B** × **C** × D × E)
							√**(D × F).		

***The Documentary stimuli (task II)***									

**The omnibus test (α = 0.050)**	√***(c > e > p)	×	√**(f > m)	√***(a > c)	√***(f > m)	√***	√***(A × **B**); √**(A × E);	√***(A × **B** × F);	√**(**B** × **C** × E × F)
							√***(A × F); √***(**B** × F);	√***(A × E × F);	
							√**(**C** × F); √***(D × F);	√**(**B** × D × F);	
							√***(E × F).	√***(**B** × E × F);	
								√***(**C** × E × F);	
								√***(D × E × F).	
***Three follow-up tests (*α*’* = *0.017)***									
(1) Emotional empathy	/	×	×	√***(a > c)	√***(f > m)	√***	√**(**B** × F); √***(E × F).	√***(D × E × F).	√*(**B** × **C** × E × F)
(2) Cognitive empathy	/	×	√**(f > m)	√**(a > c)	√***(f > m)	√***	√***(**B** × F); √*(**C** × F);	√***(**B** × E × F);	×
							√***(D × F); √***(E × F).	√***(D × E × F).	
(3) Perspective-taking	/	×	×	×	√***(f > m)	√***	√***(**B** × F); √**(D × F);	√*(**B** × **C** × F);	×
							√***(E × F).	√**(**C** × E × F).	

^⋇^For the empathy task, c, cognitive empathy; e, emotional empathy; p, perspective-taking. ^†^For the participant sex and the stimulus sex, f, female; m, male. ^∵^For the stimulus ethnicity, a, typical East Asians; c, typical Western Caucasians. ♥For the stimulus emotion, its pairwise comparison results were summarized in Supplementary Document 5. ^■^For the interaction effects, A, empathy task (i.e., emotional empathy and cognitive empathy for task I; emotional empathy, cognitive empathy, and perspective-taking for task II); **B**, **participant culture**; **C**, **participant sex**; D, stimulus ethnicity; E, stimulus sex; F, stimulus emotion. Specifically, the **participant traits are in bold**, and the “**B**×**C**” represents the culture–sex interaction effect in empathy proposed by Zhao et al. (2019). In this table, only details of significant results were presented; √, significant; ×, non-significant; /, not relevant. *p < 0.050; **p < 0.010; ***p < 0.001.

#### Omnibus Test

##### Four-way interaction

One four-way interaction, participant culture × participant sex × stimulus ethnicity × stimulus sex [*F*(1, 5,873.99) = 5.11, *p* = 0.024], was significant. Pairwise comparisons yielded the following results. First, Chinese female participants showed an ethnic out-group bias on male stimuli (i.e., Caucasian male > Asian male stimuli, *p* = 0.011, 95% CI = [0.07, 0.55]). Second, Australian male participants had the sex out-group favor (i.e., female > male stimuli) on both Caucasian stimuli (*p* = 0.037, 95% CI = [0.02, 0.55]) and Asian stimuli (*p* = 0.005, 95% CI = [0.11, 0.65]).

##### Three-way interaction

One three-way interaction was identified, namely, participant culture × stimulus ethnicity × stimulus emotion^11^ [*F*(5, 5,874.06) = 2.59, *p* = 0.024]. Results of the pairwise comparisons were threefold. First, the participant culture effects were significant on five stimuli, namely, Caucasian happiness (i.e., Australian > Chinese participant, *p* = 0.019, 95% CI = [0.10, 1.11]), Asian happiness (i.e., Australian > Chinese participant, *p* = 0.001, 95% CI = [0.33, 1.34]), Asian neutral-peacefulness (i.e., Australian > Chinese participant, *p* = 0.006, 95% CI = [0.20, 1.21]), Asian anger (i.e., Chinese > Australian participant, *p* = 0.047, 95% CI = [0.01, 1.02]), and Asian fear (i.e., Chinese > Australian participant, *p* = 0.001, 95% CI = [0.32, 1.33]). Second, the stimulus ethnicity effect on neutral-peacefulness was consistent (i.e., both were Caucasian > Asian stimuli) between Australian participants (*p* = 0.031, 95% CI = [0.03, 0.67]) and Chinese participants (*p* < 0.001, 95% CI = [0.36, 0.97]). Third, specifically, Chinese participants exhibited an ethnic in-group bias in empathy for fear (i.e., Asian > Caucasian stimuli, *p* = 0.035, 95% CI = [0.02, 0.64]) and an ethnic out-group bias in empathy for sadness (i.e., Caucasian > Asian stimuli, *p* = 0.004, 95% CI = [0.14, 0.75]).

##### Two-way interaction

Three significant two-way interactions were related to participant traits and were not qualified by the above higher-order interaction effects.

(1) Empathy task × participant culture [*F*(1, 5,873.99) = 96.45, *p* < 0.001]. Results of the pairwise comparisons revealed the following effects. First, the empathy task effects were significant for both Australian (i.e., cognitive > emotional empathy, *p* < 0.001, 95% CI = [0.31, 0.58]) and Chinese participants (i.e., emotional > cognitive empathy, *p* < 0.001, 95% CI = [0.33, 0.58]). Second, the participant culture effects were significant for both emotional empathy (i.e., Chinese > Australian participant, *p* = 0.029, 95% CI = [0.50, 0.89]) and cognitive empathy (i.e., Australian > Chinese participant, *p* = 0.042, 95% CI = [0.02, 0.85]).

(2) Empathy task × participant sex [*F*(1, 5,873.99) = 28.60, *p* < 0.001]. Pairwise comparisons of this interaction suggested the following two points. First, the empathy task effects differed between male (i.e., emotional > cognitive empathy, *p* < 0.001, 95% CI = [0.12, 0.38]) and female participants (i.e., cognitive > emotional empathy, *p* < 0.001, 95% CI = [0.12, 0.36]). Second, the participant sex effect was significant for cognitive empathy (i.e., female > male participant, *p* = 0.039, 95% CI = [0.02, 0.86]).

(3) Participant sex × stimulus emotion^1^ [*F*(5, 5,874.01) = 3.44, *p* = 0.004]. Pairwise results revealed that the participant sex effect was significant on neutral-peacefulness (i.e., female > male participants, *p* = 0.014, 95% CI = [0.12, 1.03]).

#### Follow-Up Analyses

Since the above omnibus test revealed significant interaction effects relating to the ‘empathy task’, follow-up analyses were conducted for two types of the empathy task of task I (viz., emotional empathy and cognitive empathy), separately (adjusted significance level α = 0.025).

##### Emotional empathy

###### Two-way interaction

Two significant two-way interactions were related to participant traits.

(1) Participant culture × stimulus emotion^11^ [*F*(5, 2,874.00) = 11.81, *p* < 0.001]. According to the pairwise comparisons, the participant culture effect (i.e., both were Chinese > Australian participant) was significant for both anger (*p* = 0.002, 95% CI = [0.35, 1.57]) and fear (*p* = 0.005, 95% CI = [0.27, 1.49]).

(2) Participant sex × stimulus emotion^11^ [*F*(5, 2,874.01) = 4.17, *p* < 0.001]. However, the participant sex effect was no longer significant when the pairwise comparisons were made for each stimulus emotion (all *p*s ≥ 0.158).

###### Main effect

The main effect of the stimulus sex was significant (i.e., female > male stimuli) [*F*(1, 2,874.23) = 8.61, *p* = 0.003].

##### Cognitive empathy

###### Four-way interaction

A marginally significant four-way interaction emerged; that is, participant culture × participant sex × stimulus ethnicity × stimulus sex [*F*(1, 2,873.98) = 4.24, *p* = 0.040]. According to the pairwise comparisons, Australian male participants had a significant sex out-group favor on the Asian stimuli (i.e., female > male stimuli, *p* = 0.006, 95% CI = [0.14, 0.83]).

###### Two-way interaction

There was one significant two-way interaction that related to participant traits, which was not qualified by the above four-way interaction. It was participant culture × stimulus emotion^11^ [*F*(5, 2,873.99) = 17.10, *p* < 0.001]. Pairwise comparisons indicated that the participant culture effect (i.e., Australian > Chinese participant) was significant for happiness (*p* < 0.001, 95% CI = [0.73, 1.76]) and neutral-peacefulness (*p* < 0.001, 95% CI = [0.60, 1.63]).

### State Empathy for the Documentary Stimuli

#### Omnibus Test

##### Four-way interaction

One four-way interaction was identified, namely, participant culture × participant sex × stimulus sex × stimulus emotion^1^ [*F*(5, 8,874.01) = 3.85, *p* = 0.002]. Results of its pairwise comparisons covered the following three aspects. First, the participant culture effect (i.e., all were Chinese > Australian participant) was identified with male participants on three stimuli (*p* = 0.024, 95% CI = [0.08, 1.18], for female fear; *p* = 0.003, 95% CI = [0.29, 1.39], for male surprise; *p* = 0.001, 95% CI = [0.35, 1.44], for male neutral-peacefulness) and with female participants on three stimuli (*p* = 0.002, 95% CI = [0.31, 1.35], for female fear; *p* = 0.008, 95% CI = [0.19, 1.22], for male fear; *p* = 0.029, 95% CI = [0.06, 1.10], for male surprise).

Second, the participant sex effect (i.e., all were female > male participant) was identified with Australian participants on six stimuli (*p* < 0.001, 95% CI = [0.84, 1.94], for female happiness; *p* = 0.034, 95% CI = [0.04, 1.14], for female surprise; *p* = 0.015, 95% CI = [0.13, 1.23], for male sadness; *p* = 0.014, 95% CI = [0.14, 1.24], for male anger; *p* = 0.011, 95% CI = [0.16, 1.26], for male surprise; *p* < 0.001, 95% CI = [0.67, 1.77], for male neutral-peacefulness) and also identified with Chinese participants on two stimuli (*p* = 0.023, 95% CI = [0.09, 1.12], for female happiness; *p* = 0.029, 95% CI = [0.06, 1.10], for female fear).

Third, in the aspect of the stimulus sex effect, the sex in-group favor was observed for overall empathy for happiness with both Australian male participants (i.e., male > female stimuli, *p* = 0.029, 95% CI = [0.04, 0.75]) and Australian female participants (i.e., female > male stimuli, *p* < 0.001, 95% CI = [0.39, 1.06]). Meanwhile, a sex in-group favor (i.e., female > male stimuli) on overall empathy for fear was shown by both Australian female participants (*p* = 0.029, 95% CI = [0.04, 0.71] and Chinese female participants (*p* = 0.002, 95% CI = [0.19, 0.82]). Moreover, Australian female participants had a sex out-group favor (i.e., male > female stimuli) on overall empathy for sadness (*p* < 0.001, 95% CI = [0.24, 0.92]) and anger (*p* = 0.016, 95% CI = [0.08, 0.75]). In addition, the stimulus sex effect (i.e., female > male stimuli) was consistent for the stimuli of surprise and neutral-peacefulness among all culture–sex participants groups, namely, Australian male participants (both *p*s < 0.001; 95% CI = [0.87, 1.58], for surprise; 95% CI = [1.37, 2.08], for neutral-peacefulness), Australian female participants (both *p*s < 0.001; 95% CI = [0.77, 1.45] for surprise; 95% CI = [0.54, 1.22], for neutral-peacefulness), Chinese male participants (*p* = 0.013, 95% CI = [0.09, 0.77], for surprise; *p* < 0.001, 95% CI = [0.65, 1.33], for neutral-peacefulness), and Chinese female participants (*p* = 0.002, 95% CI = [0.18, 0.82], for surprise; *p* < 0.001, 95% CI = [0.83, 1.47], for neutral-peacefulness).

##### Three-way interaction

Two significant three-way interactions were related to participant traits, and were not qualified by the above four-way interaction.

(1) Empathy task^11^. × participant culture × stimulus emotion^11^ [*F*(10, 8,874.00) = 4.80, *p* < 0.001]. Pairwise comparisons revealed that the participant culture effect (i.e., all were Chinese > Australian participant) was significant in four comparisons. They were emotional empathy for surprise (*p* = 0.001, 95% CI = [0.27, 1.10]), emotional empathy for neutral-peacefulness (*p* = 0.017, 95% CI = [0.09, 0.92]), perspective-taking for sadness (*p* = 0.021, 95% CI = [0.07, 0.90]), and perspective-taking for fear (*p* < 0.001, 95% CI = [0.98, 1.80]).

(2) Participant culture × stimulus ethnicity × stimulus emotion^11^ [*F*(5, 8,874.04) = 3.24, *p* = 0.006]. Pairwise comparisons revealed: First, the participant culture effect (i.e., all were Chinese > Australian participant) was identified on four types of stimuli; namely, Caucasian fear (*p* < 0.001, 95% CI = [0.29, 1.04]), Asian fear (*p* = 0.001, 95% CI = [0.24, 1.00]), Asian surprise (*p* = 0.049, 95% CI = [< 0.01, 0.76]), and Asian neutral-peacefulness (*p* = 0.012, 95% CI = [0.11, 0.86]). Second, in light of neutral-peacefulness, the stimulus ethnicity effect (i.e., both were Asian > Caucasian stimuli) was identified with both Australian participants (*p* = 0.003, 95% CI = [0.13, 0.62]) and Chinese participants (*p* < 0.001, 95% CI = [0.49, 0.96]). In addition, Australian participants had a significant ethnic out-group bias on happiness (i.e., Asian > Caucasian stimuli, *p* = 0.020, 95% CI = [0.05, 0.54]). In contrast, Chinese participants had a significant ethnic in-group bias on sadness (i.e., Asian > Caucasian stimuli, *p* < 0.001, 95% CI = [0.16, 0.63]).

#### Follow-Up Analyses

Since the above omnibus test revealed significant interaction effects on the ‘empathy task’, follow-up analyses were conducted for three types of the empathy task of task II (viz., emotional empathy, cognitive empathy, and perspective-taking), separately (adjusted significance level α = 0.017).

##### Emotional empathy

###### Four-way interaction

One four-way interaction was marginally significant, which was participant culture × participant sex × stimulus sex × stimulus emotion^11^ [*F*(5, 2,874.01) = 2.66, *p* = 0.021]. Pairwise comparisons suggested: First, the participant culture effect (i.e., Chinese > Australian participant) was significant on male surprise with both male and female participants (*p* = 0.015, 95% CI = [0.21, 1.89], for male participant; *p* = 0.016, 95% CI = [0.18, 1.78], for female participant), and was also significant on male neutral-peacefulness with male participants (*p* = 0.002, 95% CI = [0.49, 2.17]). Second, the participant sex effect (i.e., female > male participant) was significant with Australian participants on both female happiness (*p* = 0.006, 95% CI = [0.34, 2.02]) and male neutral-peacefulness (*p* < 0.001, 95% CI = [0.74, 2.42]). Third, the stimulus sex effect (i.e., all were female > male stimuli) was significant with three culture–sex participant groups, namely, Australian male participants (*p* = 0.003, 95% CI = [0.26, 1.24], for surprise; *p* < 0.001, 95% CI = [0.78, 1.76], for neutral-peacefulness), Australian female participants (*p* = 0.002, 95% CI = [0.28, 1.21], for happiness; *p* = 0.008, 95% CI = [0.16, 1.10], for surprise), and Chinese female participants (*p* = 0.005, 95% CI = [0.19, 1.07], for fear; *p* < 0.001, 95% CI = [0.33, 1.21], for neutral-peacefulness).

##### Cognitive empathy

###### Three-way interaction

One significant three-way interaction was participant culture × stimulus sex × stimulus emotion^11^ [*F*(5, 2,874.03) = 4.14, *p* < 0.001]. The following effects were significant according to the pairwise comparisons. First, the participant culture effect was significant on both male anger (i.e., Australian > Chinese participant, *p* = 0.001, 95% CI = [0.25, 1.05]) and male surprise (i.e., Chinese > Australian participant, *p* = 0.006, 95% CI = [0.17, 0.97]). Second, the stimulus sex effect (i.e., all were female > male stimuli, all *p*s < 0.001) was observed with both Australian participants (95% CI = [1.26, 1.91], for surprise; 95% CI = [1.26, 1.91], for neutral-peacefulness) and Chinese participants (95% CI = [0.51, 1.14], for surprise; 95% CI = [0.94, 1.55], for neutral-peacefulness).

###### Two-way interaction

One two-way interaction with participant traits was not qualified by the above higher-order interaction effects; that is, participant sex × stimulus emotion^11^ [*F*(5, 2,874.01) = 2.80, *p* = 0.016]. According to the pairwise comparisons, the participant sex effect (i.e., all were female > male participant) was significant for four emotions, namely, happiness (*p* < 0.001, 95% CI = [0.27, 0.94]), anger (*p* = 0.006, 95% CI = [0.14, 0.80]), surprise (*p* < 0.001, 95% CI = [0.27, 0.93]), and neutral-peacefulness (*p* = 0.003, 95% CI = [0.17, 0.83]).

##### Perspective Taking

###### Three-way interaction

Two significant interactions were found.

(1) Participant sex × stimulus sex × stimulus emotion^11^ [*F*(5, 2,874.00) = 3.57, *p* = 0.003]. Pairwise comparisons indicated: First, the participant sex effect (i.e., both were female > male participant) was significant on both female happiness (*p* < 0.001, 95% CI = [0.81, 2.04]) and female surprise (*p* = 0.009, 95% CI = [0.20, 1.43]). Second, for both male and female participants, the stimulus sex effect (i.e., all were female > male stimuli) was significant on both surprise (*p* = 0.006, 95% CI = [0.19, 1.14], for male participant; *p* < 0.001, 95% CI = [0.64, 1.54], for female participant) and neutral-peacefulness (both *p*s < 0.001; 95% CI = [1.10, 2.04], for male participant; 95% CI = [0.83, 1.72], for female participant). In addition, male participants showed a sex in-group favor on happiness (i.e., male > female stimuli, *p* < 0.001, 95% CI = [0.42, 1.36]). Meanwhile, female participants had a sex out-group favor on sadness (i.e., male > female stimuli, *p* = 0.002, 95% CI = [0.24, 1.13]).

(2) Participant culture × participant sex × stimulus emotion^11^ [*F*(5, 2,874.01) = 2.60, *p* = 0.024]. Pairwise comparisons for this marginal significant interaction showed: First, the participant culture effect on fear was significant with both male participants (i.e., Chinese male > Australian male participant, *p* = 0.010, 95% CI = [0.25, 1.76]) and female participants (i.e., Chinese female > Australian female participant, *p* < 0.001, 95% CI = [1.06, 2.49]). Second, the participant sex effect on happiness was significant with Australian participants (i.e., Australian female > Australian male participant, *p* = 0.007, 95% CI = [0.28, 1.79]).

## Discussion

In this study, the culture–sex interaction effect in empathy was studied with Australian and Chinese participants. Moreover, this interaction effect was identified on both trait and state empathy. For trait empathy, the current observation was consistent with previous findings (Melchers et al., 2015; Zhao et al., 2019). For state empathy, the culture–sex interaction effect further interacted with stimulus traits (e.g., stimulus ethnicity, stimulus sex, and stimulus emotion), resulting in three- or four-way interactions (see Table 5). Follow-up analyses of the higher-order interactions revealed that the impacts of stimulus traits varied among the culture–sex participant groups (i.e., Australian female, Australian male, Chinese female, and Chinese male). To conclude, the current results support the theory of culture–sex interaction effect in empathy (Zhao et al., 2019). Furthermore, the current results highlight that beyond the fundamental culture–sex interaction effect in empathy, there could be more intriguing interactions across participant traits and stimulus features.

### Trait Empathy

The culture–sex interaction effect emerged as a clear trend in terms of trait empathy (see Table 4). This finding is in line with that of Zhao et al. (2019), who evaluated trait empathy with Australian Caucasian (*n* = 196) and Chinese Han (*n* = 211) university students. Specifically, in both the current and the previous study (Zhao et al., 2019), the cultural differences in trait empathy were significant in female participants (i.e., Australian female > Chinese female participant) but not in male participants. Furthermore, sex differences in trait empathy were only significant with Australian participants (i.e., Australian female > Australian male participant) but not with Chinese participants. Zhao et al. (2019) proposed that the culture–sex interaction in trait empathy might be germane to social expectations for emotional expressions. Generally, Western cultures encourage females to externalize their emotions more than males (i.e., the so-called emotional female and rational male; Merten, 2005). In contrast, honoring Confucius’ Golden Mean philosophy, both Chinese males and Chinese females are supposed to avoid being either extremely emotional or extremely restrained (Huang, 2006; Zhao et al., 2019), resulting in a diminished sex difference in empathy (also see Zhao et al., 2020). Nevertheless, it should be noted that the above relationship between empathy and social expectations is only a theoretical proposal by Zhao et al. (2019), and future empirical studies are necessary to verify this proposal.

### State Empathy

The current state empathy results were more complex, spanning significant two-, three-, and four-way interactions (see Table 5). For example, there were four-way interactions on overall and cognitive empathy for NimStim stimuli (i.e., participant culture × participant sex × stimulus ethnicity × stimulus sex), four-way interactions on overall and emotional empathy for the documentary stimuli (i.e., participant culture × participant sex × stimulus sex × stimulus emotion), as well as one three-way interaction on perspective-taking of the documentary stimuli (i.e., participant culture × participant sex × stimulus emotion).

#### The Culture–Sex Interaction Effect

Within each of the aforementioned three- and four-way interactions, there is a culture–sex interaction effect. Moreover, these three- and four-way interactions covered all forms of state empathy examined in this study (i.e., overall empathy, emotional empathy, cognitive empathy, and perspective-taking). On the one hand, the current findings suggest that culture–sex interaction effects in empathy are not restricted to trait empathy (e.g., Zhao et al., 2019) but can expand to state empathy. On the other hand, the current results are similar to the findings of Zhao et al. (2019), suggesting that the culture–sex interaction is significant for inclusive components of empathy (see Melchers et al., 2015 and Lachmann et al. (2018), both of them found the interaction was not significant on cognitive trait empathy). It is worth mentioning that Schmitt (2015) had a theory of “culturally variable sex difference”; as per Schmitt (2015), the culture–sex interaction effect could be a non-negligible phenomenon in a broad range of social and psychological subjects in addition to empathy. Therefore, the culture–sex interaction effect deserves attention from future cross-cultural researchers of sociology and psychology.

However, the culture–sex interaction effect has been ignored by most of the previous investigators of the Western–Asian cultural difference in trait and state empathy (see Tables 1–3). As noted by Zhao et al. (2019), the culture–sex interaction effect could be an explanation for the inconsistent results among the publications (see Tables 1–3). Moreover, Zhao et al. (2019) proposed that the magnitude of the Western–Asian cross-cultural differences in trait empathy could be enlarged along with the female ratio of a sample (i.e., a positive correlation with the female%). Both the current study and Zhao et al. (2019) presented supporting evidence for the above notion since the effect size of the cultural difference in trait empathy tends to be larger for female participants relative to male participants.

#### Participant Culture Effect

Referencing the results of culture–sex interaction in trait empathy (Zhao et al., 2019), the Australian females should be the most empathic among the four culture–sex participant cohorts. Nevertheless, the current findings for state empathy revealed a different trend; that is, the advantages and disadvantages of state empathy are relatively counterbalanced for the participant groups. First, in light of the NimStim stimuli (i.e., task I), Australian participants expressed more cognitive empathy for positive and neutral stimuli (i.e., happiness and neutral-peacefulness). In contrast, Chinese participants reported more emotional empathy for negative emotions (i.e., anger and fear). Second, in light of the documentary stimuli (i.e., task II), the Chinese participants commonly expressed more empathy (i.e., overall empathy, emotional empathy, cognitive empathy, and perspective-taking) than Australian participants. However, Australian participants specifically reported more cognitive empathy for stimuli of male anger than Chinese participants did.

The inconsistency among the findings of trait empathy and state empathy (for NimStim and for documentary stimuli) is intriguing and can be explained by a range of factors. The first factor is social expectation. On the one hand, as per Zhao et al. (2019), Australian females’ higher self-evaluated trait empathy could be largely due to the social expectation placed on them. However, the impact of social expectation on the computer-based evaluations (i.e., state empathy) could be weaker than that on self-report evaluations (i.e., trait empathy) (Baez et al., 2017). More importantly, in the current study, participants were explicitly required to answer each state empathy question according to their inner feelings rather than social justice (see the section “Materials and Methods”). This instruction might have minimized the impact of social expectation on the state empathy tasks. On the other hand, Chinese traditional cultures (e.g., Confucianism and Taoism) honor humility and modesty in individuals (Lin et al., 2018). Hence, Chinese participants could downplay themselves while answering the trait empathy items (i.e., the items enquire ‘‘how good the participant is in empathy’’)^12^ but might be more objective during responding to state empathy questions (i.e., the questions ask ‘‘how much the participant felt for a given stimulus’’)^13^. Therefore, Chinese participants may seem to be less empathic than Australian participants in light of trait empathy (i.e., the self-report scales assessed) but not state empathy (i.e., the computer-tasks evaluated).

The second factor is the background information of the stimuli. The current results suggest that when the emotional background information was withheld (i.e., the NimStim stimuli), Australian participants had higher cognitive empathy for neutral and happy stimuli, while Chinese participants showed more emotional empathy for negative emotions. This observation was in agreement with the distinct Asian and Western cultural requirements of emotional expression and suppression. Generally, in Asian societies, negative emotions are expected to be masked (e.g., by a neutral or smiling face) for maintaining interpersonal harmony (Wei et al., 2013). This social rule is different from Western societies, in which externalizing emotions is accepted as an honest way to express oneself (Gross and John, 2003; Murata et al., 2012). Consequently, since childhood, Chinese individuals have been trained to decode others’ emotions according to contextual information, as well as trained to be alert to others’ subtle emotional downturns (i.e., watch the “face colors”) (Wang, 2001). Therefore, emotional understanding (i.e., cognitive empathy) for neutral and happy faces without emotional background information could be a challenge for Chinese participants (i.e., as per the Chinese culture, a neutral or happy face by itself could indicate neutral, happy, or masked negative feelings). However, the empathic sensitivity (i.e., emotional empathy) for negative emotions might be more intense for the Chinese than Australian participants (i.e., due to the necessity of watching others’ “face colors” in Chinese society) (e.g., Wang, 2001).

In contrast, when the background information was given (i.e., the documentary stimuli), empathy for most of the emotions was promoted for Chinese participants. One exception was the cognitive empathy for the stimuli of male anger. Anger is an intense emotion that disturbs the harmony of interpersonal relationships (de Greck et al., 2012). Influenced by the Confucian Golden Mean philosophy, the Chinese may value social harmony much more than Westerners (Drummond and Quah, 2001; de Greck et al., 2012; Liu, 2014). In light of Chinese culture, expressing anger could be labeled as lacking in self-control (Kornacki, 2001; Kong et al., 2020). In contrast, for Westerners, sincerely expressing emotions could be deemed as a way to enhance interpersonal understanding (Gross and John, 2003; Murata et al., 2012). Moreover, de Greck et al. (2012) decoded the neurological basis of Western–Asian cultural differences in empathy for anger. They found that facing ethnic in-group anger, German participants had more brain activation in the cognitive empathy-related brain regions (i.e., the inferior temporal gyrus and middle insula). In contrast, Chinese participants showed more brain activation in the emotional regulation and personal distress-related brain region (i.e., the dorsolateral prefrontal cortex). de Greck et al. (2012) claimed that the Western participants might try to understand the anger; meanwhile, the Chinese participants might attempt to inhibit their aversive feelings stirred up by the anger. Noticeably, some previous researchers of cultural differences in empathy (see Tables 2, 3) adopted the concepts of “negative emotions” or “suffering” (i.e., mixed negative emotions) as emotional stimuli. However, the current results highlight that the participants’ cultural differences in empathy can be qualified by the subtypes of negative emotions.

#### Ethnic Group Bias

In this study, the dominant trend of ethnic group bias in state empathy was the ethnic in-group bias for negative emotions together with the ethnic out-group bias for positive emotions. These findings were in line with our hypothesis (see the section “Introduction”) as well as the previous observation by Neumann et al. (2013). Specifically, the current Chinese participants exhibited ethnic in-group biases on overall empathy (i.e., the holistic concept of emotional and cognitive empathy) for fear (NimStim stimuli) and sadness (documentary stimuli).

In contrast, the current Australian participants expressed an ethnic out-group bias on overall empathy for happiness (documentary stimuli). These findings cannot be fully explained by either the theory of in-group familiarity (Cao et al., 2015) or the one of out-group hate (Avenanti et al., 2010). Instead, as discussed in the Introduction section, being concerned about in-groups in need (i.e., the in-group bias for negative emotions) and out-groups in a triumphant mood (i.e., the out-group bias for happiness) could be two facets of the “reciprocal altruism” (Trivers, 1971; Mathur et al., 2010).

Nevertheless, two exceptions of ethnic group bias in state empathy were identified with the current Chinese participants (both for NimStim stimuli). First, the Chinese participants showed an ethnic out-group bias for the NimStim sadness. Sadness may be perceived as a symbol of powerlessness and low self-esteem (Merten, 2005); an exposure of one’s weakness in front of others without a good reason could be interpreted by Chinese people as “losing face” (i.e., a Chinese word, describing the feeling of embarrassment and shame for oneself as a consequence of unsuitable conduct; Ho, 1976; Zhang et al., 2011). Trommsdorff et al. (2007) coined the term “non-acting” to explain the same situation. They stated that in cultures that discourage emotional externalization, individuals might purposely inhibit their reactions to an emotional person so as to “save that person’s face” (Trommsdorff et al., 2007). Therefore, the current Chinese participants might refrain from empathy toward the Asian characters expressing sadness without a good reason (i.e., NimStim stimuli), leading to the out-group bias. However, as long as an emotional background was given for the sadness of the documentary stimuli (e.g., an earthquake or bushfire ruin), the ethnic group bias of the current Chinese participants turned into an ethnic in-group bias.

Second, there was a four-way interaction (i.e., participant culture × participant sex × stimulus ethnicity × stimulus sex) on the overall empathy for NimStim stimuli. Further examination of the four-way interaction showed an ethnic out-group bias with the Chinese female participants on NimStim male stimuli. The reasons for the ethnic out-group bias could be still due to the non-acting strategy (Trommsdorff et al., 2007). Relatively, Western cultures provide more freedom for individuals to express their emotions, while Asian cultures value emotion regulation more (Davis et al., 2012; Wei et al., 2013). Moreover, with Chinese and American participants, Davis et al. (2012) found that Chinese male participants expressed the highest emotion regulation, which was in line with their concern that the social pressure on moderating emotions was stronger for Chinese males than the other culture–sex participant groups (i.e., a culture–sex interaction effect in emotion regulation). Hence, the current Chinese female participants might adopt the non-acting strategy to specifically “save the face” of the NimStim Asian male over the NimStim Caucasian male (Trommsdorff et al., 2007). This turned out to be the Chinese female participants’ ethnic out-group bias in empathy. Nonetheless, when the emotional background was illustrated with the emotion (i.e., the documentary stimuli), the ethnic out-group bias for male stimuli was absent from the Chinese female participants. The above results stress that the ethnic group bias may vary among the culture–sex participant groups, which can be moderated by the availability of the background information; however, these possibilities were overlooked by previous researchers (Tables 2, 3).

#### Sex Group Favor

Sex group favor in empathy was not examined in previous studies summarized in Tables 1–3. The current results revealed that the main sex group favor was biased to female (i.e., female > male stimuli, see Table 5). This main favor is consistent with a common social consensus, namely, females are more vulnerable and should be treated with extra consideration (i.e., the “ladies first” ideology) (Tuleja, 2012). Nevertheless, some minor variations on the sex favor effect could still be identified among the four culture–sex participant groups. First, in light of the NimStim stimuli, the sex group favor (i.e., female > male stimuli) was only significant with Australian male participants (i.e., the overall empathy for both Caucasian and Asian stimuli, as well as cognitive empathy for Asian stimuli), but not with the other three culture–sex participant groups. Second, in light of the documentary stimuli, the main sex group favor (i.e., female > male stimuli) was identifiable with all culture–sex participant groups. However, this ‘ladies first’ favor in empathy for the documentary stimuli tended to be stronger for the Australian than Chinese participants; this result also supported the notion that sex differentiation is more pronounced in Western than in Asian cultures (Zhao et al., 2019).

Third, the opposite sex group favor (i.e., the “alpha male” ideology) was also presented in the current results, particularly with the Australian participants. On the one hand, Australian male participants expressed more overall empathy for male happiness of the documentary stimuli (i.e., a male runner in the marathon) than the female ones (i.e., a bride in the wedding ceremony). Intriguingly, toward the same stimuli, the Australian female participants’ sex favor on the overall empathy was biased to female (i.e., the bride’s happiness > the male runner’s happiness). In contrast, Chinese female and Chinese male participants showed non-significant sex favor on the overall empathy for happiness (i.e., the bride’s happiness = the male runner’s happiness). Besides further stressing that sex differentiation can be more polarized in Western than in Asian cultures, the above results are in line with the stereotype of Australian males (i.e., the ‘Sporting Manhood in Australia’; Adair et al., 1997, 1998).

On the other hand, Australian female participants’ sex group favors on overall empathy for the documentary stimuli of anger and sadness were biased to male (i.e., male > female stimuli; see Supplementary Document 1 for the stimuli’s background information). Teague (2014) evaluated empathic accuracies with three ethnic groups of Americans (viz., Caucasian, African, and Chinese). Teague found that relative to the male participants, the female participants of all three ethnic groups tended to be more sensitive to negative emotions (e.g., anger and sadness) expressed by Caucasian characters (i.e., the main ethnicity of the country) (Teague, 2014, see pp. 107–108). Moreover, relative to African male participants, the African female participants were hypersensitive to in-group anger and sadness (i.e., expressed by African characters). In contrast, Chinese female and Chinese male participants’ reactions toward in-group anger and sadness (i.e., expressed by Chinese characters) were relatively similar. Results of Teague (2014) and the current study imply that sex group difference and sex group favor in empathy for negative emotions may be relevant to social vulnerability, and the female vulnerability may be more obvious in Western than Asian societies. Nevertheless, since Teague (2014) did not split the stimuli according to stimulus sex, whether females in Western societies were specifically sensitive to male negative emotions was not definitive. Nevertheless, the current results indicate that Western females may be more empathic toward male anger and sadness than female ones. The sex group favor in empathy, especially the sex favor against common consensus (i.e., the alpha male ideology), is worthy of further investigation.

### Limitations and Further Studies

The current study has several limitations. First, the sample size was small. Conclusions regarding the interaction effects in state empathy need to be replicated based on a larger sample size. Second, only university students were recruited in this study, and hence, the current findings might not be extended to the general populations of Australia and China. Third, in this study, the ethnic group bias and sex group favor were only explored in terms of state empathy but not trait empathy (i.e., the EQ and IRI items do not examine these phenomena). Further researchers might consider investigating these phenomena in trait empathy using self-report questionnaires. However, it should be noted that participants can interpret questions regarding ethnic group bias and sex group favor as tapping into racism and sexism. Consequently, participants may respond to these questions according to social desirability (i.e., without racism and sexism). Fourth, it should be noted that the empathic accuracies of some emotions (e.g., fear, surprise, and neutral-peacefulness) were low in the current study (see Supplementary Document 1). Result interpretations for these emotions with a low empathic accuracy should be done with care. Fifth, questions of state empathy presented in the current computer-based tasks could still be categorized as subjective (e.g., “I felt _____the feeling of the main character”) although they were comparatively more objective than the self-report items of trait empathy (i.e., the EQ and IRI items). The culture–sex interaction, ethnic group bias, and sex group favor effects ought to be verified by more objective techniques, such as brain imaging or physiological measurements (see Neumann and Westbury, 2011; Neumann et al., 2015). Sixth, to date, the culture–sex interaction effect in empathy with adult participants has been identified by Melchers et al. (2015) (i.e., Germans vs. Chinese), Zhao et al. (2019) (i.e., Australians vs. Chinese), as well as the current study (i.e., Australians vs. Chinese). It is noteworthy that the Asian participants of these three studies were all Chinese. Thereby, it is essential to verify in further investigations whether the culture–sex interaction in empathy can be generalized to other Asian cultures; in other words, whether the culture–sex interaction effect is a common phenomenon of the Western–Asian contrast or is a specific term to the Western–Chinese contrast^14^.

In addition, some limitations of the current computer-based tasks of state empathy should be elaborated. Firstly, the current participants’ attitudes toward the other ethnicity (e.g., whether they had out-group hate) were not collected. The current authors deemed that out-group hate might not be a serious issue in the current case since both Australian and Chinese participants expressed the ethnic out-group bias in state empathy. Nevertheless, it is highly recommended for further investigators to record participants’ attitudes toward other ethnicities to elaborate on this topic. Secondly, each component of state empathy was evaluated by a single item (e.g., “I felt _____ the feeling of the main character. 1 = *not at all* to 9 = *very strongly*”, for emotional state empathy). The single-item design (i.e., also used by all previous investigations, see Tables 2, 3) could be criticized as not sufficiently reliable to capture the relatively stable psychological traits of empathy. A multi-item evaluation of state empathy ought to be considered in future investigations. Thirdly, participants’ state empathy could be confounded by stimulus traits (e.g., age, clothing, and attractiveness of the character), which were not controlled in the current examinations. Fourthly, we did not directly compare the results of state empathy for NimStim stimuli with that for the documentary stimuli (i.e., tasks I and II, respectively) to evaluate the impact of background information on empathy. It should be noted that the stimuli of tasks I and II were different in several important aspects, including the availability of background information, the facial expressivity of the main characters, and more importantly, whether the characters expressed an emotion naturally. To evaluate the impact of background information on empathy, a future investigation with better-manipulated stimuli is necessary (i.e., an identical facial expression with different background information). Fifthly, regarding the stimuli of task II, we chose documentary photos of naturally expressed emotions with matched background information across Western and Asian stimuli (see details in Supplementary Document 1). Alternatively, researchers can do a computer manipulation on the facial expressions of those main characters to get a standard facial expression across Western and Asian stimuli. However, we are concerned that computer-modified facial expressions may change the social meaning and the biological validity of the stimuli. It is because emotional expressivity naturally differs between cultures (Rychlowska et al., 2015). Under the same situation, Westerners’ facial expressions could be more exaggerated than Asians’ (e.g., laughing or smiling at their wedding party). Hence, a standard happy face deemed so by Westerners could seem ecstatic to Asians. Therefore, we recommend documentary photos (i.e., naturally expressed emotions) rather than computer-manipulated ones. Finally, it should be stressed that due to the small sample size, the current investigation may not provide enough statistical power to reveal all subtle interaction effects on state empathy. Further investigation with a larger sample size is highly recommended.

## Conclusion

The current study explored the culture–sex interaction effect for both trait and state empathy with Australian and Chinese participants. In line with previous findings, the two-way interaction emerged as a reliable effect for trait empathy. Moreover, this two-way interaction effect was also presented on state empathy through higher-order interaction effects (i.e., three- and four-way interactions). On the one hand, the culture–sex interaction effect on trait empathy revealed that the sex difference in empathy was larger for Australian than Chinese participants, while cultural difference in empathy was larger for female than male participants. On the other hand, three- and four-way interactions on state empathy highlighted the dynamic nature of empathy (i.e., its sensitivity to traits of both participant and stimulus). It is noteworthy that due to a neglect of the culture–sex and higher-order interaction effects, previous conclusions regarding the cultural impacts on empathy could be biased (as summarized in Tables 1–3). These important interaction effects in empathy, especially the culture–sex interaction effect, are worthy of further investigations.

## Data Availability Statement

The datasets generated for this study are available on request to the corresponding author.

## Ethics Statement

The studies involving human participants were reviewed and approved by the Griffith University Human Research Ethics Committee and East China Normal University Committee on Human Research Protection.

## Author Contributions

QZ collected the data, conducted the data analysis, and wrote the manuscript. DN designed the study and wrote the manuscript. CY designed the study and participated in the data collection and manuscript writing. SD participated in the manuscript writing. DS designed the study and wrote the manuscript. All authors contributed to the article and approved the submitted version.

## Conflict of Interest

The authors declare that the research was conducted in the absence of any commercial or financial relationships that could be construed as a potential conflict of interest.

## Footnotes

1 Based on Australian and Chinese female participants, Zhao et al. (2019) found that both independent and interdependent self-construals could be mediators of the prediction from culture to the trait empathy scores (i.e., maintaining a balanced self-other distance could be essential for females to obtain a higher score on trait empathy). In contrast, for male participants, only interdependent self-construal was a mediator (i.e., seeking a closer self-other relationship might be indispensable for males to reach a higher score on trait empathy) (Zhao et al., 2019).

2 Melchers et al. (2015) identified significant culture–sex interactions on emotional and overall empathy, but not cognitive empathy. They also reported that the sex differences in the trait empathy scores (i.e., emotional and overall empathy) were larger for the German than Chinese participants. Nevertheless, they did not conduct an elaborate follow-up analysis, and some details of their interactions were unidentifiable (Melchers et al., 2015).

3 Using G^∗^power, the current authors evaluated the sample size required for conducting an ANOVA test (fixed effects, special, main effects, and interactions; numerator *df* = 1; the number of groups = 4; according to a medium to large effect size *f* = [0.25 to 0.37]; α = 0.05; a power of 0.80). The recommendation for the overall sample size was 60 to 128.

4 The survey platforms were LimeSurvey.org for Australian participants and Sojump.com for Chinese participants.

5 The NimStim stimuli were the same group of models expressing different emotions. In the current investigation, four NimStim models (i.e., Model #09, #19, #20, and #45, who were one Caucasian female, one Asian female, one Caucasian male, and one Asian male, respectively) were selected.

6 For the stimuli of task II, the current authors matched the background information, pair by pair, between stimuli of Caucasian and Asian characters with the same sex. For example, in the stimulus set, there was a stimulus of a Caucasian bride at her wedding party as well as a stimulus of an Asian bride at her wedding party (see more information in Supplementary Document 1).

7 The four pictures were IAPS #2377, #2102, #6313, and #4598.

8 The Internet pictures can be acquired from the current corresponding author.

9 A group of *post hoc* and follow-up analyses was planned for the omnibus tests of ACC, but the current results revealed no significant interaction effect on the ACC (see the section “Results”).

10 The omnibus tests of state empathy (i.e., both tasks I and II) mirrored the ANOVA of the EQ (i.e., the overall empathy). In contrast, the follow-up analyses (i.e., both tasks I and II) mirrored the ANOVAs of the IRI subscales (i.e., the individual components of empathy, separately).

11 In light of the word limitation, pairwise comparisons for all stimulus emotion effects and one subsequent empathy task effect are presented in Supplementary Document 5.

12 Item examples of trait empathy (i.e., EQ and IRI) are “I am good at predicting how someone will feel.” EQ#25 and “I would describe myself as a pretty soft-hearted person.” IRI#22.

13 Question examples of state empathy (i.e., computer tasks I and II) are “I felt _____ the feeling of the main character. 1 = *not at all* to 9 = *very strongly*,” “I understood _____ the situation of the main character. 1 = *not at all* to 9 = *very fully*,” and “I can _____ imagine myself in the situation of the main character. 1 = *not at all* to 9 = *very easily*.”

14 Nevertheless, Trommsdorff et al. (2007) examined the culture–sex interaction effect in empathy with children recruited from kindergartens of four countries (i.e., Germany, Israel, Indonesia, and Malaysia). They found that the sex difference (i.e., female > male participant) in state empathy (i.e., they used the term “sympathy”) was significant with the German group but not with the other three cultural groups.
