# Supplementary material for: Culture, Sex, and Group-Bias in Trait and State Empathy
Source: Front Psychol. 2021 Apr 28;12:561930. doi: 10.3389/fpsyg.2021.561930 (PMC8113867; doi:10.3389/fpsyg.2021.561930)
Supplement: Supplementary file 1 [file Table_1.docx]

Culture, Sex, and Group-Bias in Trait and State Empathy

**Supplementary Document 1**

**Stimuli Validity and Description**

This document provides information on the stimuli validities of the current computer-based Task I (i.e., the 24 NimStim stimuli) and Task II (i.e., the 24 Documentary Stimuli). To assess the NimStim stimuli, researchers should contact the corresponding author of Tottenham et al. (2009). For the Documentary stimuli, three resources were used; namely, the International Affective Picture System (IAPS; Lang, Bradley, & Cuthbert, 1999), Neumann, Boyle, and Chan (2013), and the internet. Referencing the resource of each Documentary stimulus (see Supplementary Tables 1.10 of this document), these stimuli should be either downloaded from the IAPS (Lang et al., 1999), requested from the corresponding author of Neumann et al. (2013) and the current study (Dr. Qing Zhao, [zhaoqing-1@126.com](mailto:zhaoqing-1@126.com)).

In this document, the following information is presented:

(1) Stimulus validity of emotional intensity (i.e., the question was “How strongly was the main character feeling the emotion? 1 = *not at all* to 9 = *very strongly*”) for NimStim stimuli (see Supplementary Table 1.1) and for Documentary stimuli (see Supplementary Table 1.2);

(2) Stimulus validity of emotional valence (i.e., the question was “How negative or positive did the picture seem? 1 = *very negative* to 9 = *very positive*”) for NimStim stimuli (see Supplementary Table 1.3) and for Documentary stimuli (see Supplementary Table 1.4);

(3) Stimulus validity of emotional arousal (i.e., the question was “Viewing the picture, I felt _____. 1 = *very calm/relaxed* to 9 = *very aroused/jittery*”) for NimStim stimuli (see Supplementary Table 1.5) and for Documentary stimuli (see Supplementary Table 1.6);

(4) Stimulus validity of emotional distress (i.e., the question was “Viewing the picture, I felt _____. 1 = *very at ease/comfortable* to 9 = *very distressed*”) for NimStim stimuli (see Supplementary Table 1.7) and for Documentary stimuli (see Supplementary Table 1.8);

(5) The stimulus description and stimulus validity of empathic accuracies (ACC) (i.e., the question was “What was the main emotion that the main character was feeling? 1 = *Happiness*, 2 = *Surprise*, 3 = *Neutral*, 4 = *Fear*, 5 = *Anger*, and 6 = *Sadness*”) for NimStim stimuli (see Supplementary Table 1.9) and for Documentary stimuli (see Supplementary Table 1.10).

| Supplementary Table 1.1 | | | | | | | | | | | | | | | | | | | |
| --- | --- | --- | --- | --- | --- | --- | --- | --- | --- | --- | --- | --- | --- | --- | --- | --- | --- | --- | --- |
| *Emotional Intensity for NimStim Stimuli Based on the Current Participants* | | | | | | | | | | | | | | | | | | | |
| Stimulus information | | | | |  | Emotional Intensity^*^ | | | | | | | | | | | | | |
| No. | NimStim Code | Sex^†^ | Ethnicity^∵^ | Emotion^♥^ |  | Australian female  (*n* = 32) | |  | Australian male  (*n* = 29) | |  | Chinese female  (*n* = 36) | |  | Chinese male  (*n* = 32) | |  | Overall  (*n* = 129) | |
|  |  |  |  |  |  | *M* | *SD* |  | *M* | *SD* |  | *M* | *SD* |  | *M* | *SD* |  | *M* | *SD* |
| 1 | 09F_HA_O | F | W | Ha |  | 8.34 | 0.87 |  | 7.69 | 1.00 |  | 7.11 | 1.39 |  | 6.97 | 1.40 |  | 7.51 | 1.31 |
| 2 | 20M_HA_O | M | W | Ha |  | 7.00 | 1.50 |  | 6.79 | 1.32 |  | 6.50 | 1.40 |  | 6.19 | 1.42 |  | 6.61 | 1.43 |
| 3 | 19F_HA_O | F | A | Ha |  | 7.81 | 1.67 |  | 7.17 | 1.14 |  | 6.39 | 1.69 |  | 6.81 | 1.12 |  | 7.02 | 1.53 |
| 4 | 45M_HA_O | M | A | Ha |  | 8.16 | 1.59 |  | 7.83 | 0.97 |  | 7.22 | 1.68 |  | 7.03 | 1.73 |  | 7.54 | 1.59 |
| 5 | 09F_AN_O | F | W | An |  | 7.59 | 1.29 |  | 7.38 | 1.37 |  | 7.61 | 1.23 |  | 7.94 | 0.91 |  | 7.64 | 1.21 |
| 6 | 20M_AN_O | M | W | An |  | 7.87 | 1.70 |  | 7.72 | 1.39 |  | 8.00 | 1.17 |  | 7.84 | 1.17 |  | 7.87 | 1.35 |
| 7 | 19F_AN_O | F | A | An |  | 8.66 | 0.87 |  | 8.24 | 0.95 |  | 8.31 | 0.89 |  | 8.09 | 1.53 |  | 8.33 | 1.10 |
| 8 | 45M_AN_O | M | A | An |  | 8.50 | 0.84 |  | 7.93 | 1.31 |  | 8.11 | 0.98 |  | 8.03 | 1.03 |  | 8.15 | 1.05 |
| 9 | 09F_SA_C | F | W | Sa |  | 7.13 | 1.70 |  | 6.10 | 1.70 |  | 6.58 | 1.61 |  | 5.47 | 1.72 |  | 6.33 | 1.77 |
| 10 | 20M_SA_C | M | W | Sa |  | 7.88 | 1.18 |  | 6.72 | 1.62 |  | 7.22 | 1.31 |  | 6.25 | 1.85 |  | 7.03 | 1.61 |
| 11 | 19F_SA_C | F | A | Sa |  | 7.19 | 1.38 |  | 6.76 | 1.48 |  | 6.03 | 1.80 |  | 5.56 | 1.79 |  | 6.36 | 1.73 |
| 12 | 45M_SA_C | M | A | Sa |  | 6.91 | 1.67 |  | 6.17 | 1.63 |  | 5.78 | 1.94 |  | 5.44 | 1.87 |  | 6.06 | 1.85 |
| 13 | 09F_FE_O | F | W | Fe |  | 6.44 | 1.48 |  | 6.07 | 1.49 |  | 6.92 | 1.46 |  | 6.37 | 1.68 |  | 6.47 | 1.54 |
| 14 | 20M_FE_O | M | W | Fe |  | 6.13 | 1.74 |  | 6.03 | 1.74 |  | 5.86 | 1.61 |  | 5.81 | 1.51 |  | 5.95 | 1.63 |
| 15 | 19F_FE_O | F | A | Fe |  | 7.94 | 1.05 |  | 7.10 | 1.05 |  | 8.06 | 0.86 |  | 7.75 | 1.22 |  | 7.74 | 1.09 |
| 16 | 45M_FE_O | M | A | Fe |  | 8.34 | 0.87 |  | 7.76 | 1.12 |  | 8.22 | 1.07 |  | 8.25 | 0.76 |  | 8.16 | 0.98 |
| 17 | 09F_SP_O | F | W | Su |  | 7.28 | 1.46 |  | 6.90 | 1.11 |  | 6.97 | 1.30 |  | 6.84 | 1.46 |  | 7.00 | 1.34 |
| 18 | 20M_SP_O | M | W | Su |  | 6.50 | 1.68 |  | 6.14 | 1.33 |  | 5.72 | 1.41 |  | 5.72 | 1.35 |  | 6.01 | 1.47 |
| 19 | 19F_SP_O | F | A | Su |  | 7.62 | 1.07 |  | 6.62 | 1.18 |  | 7.19 | 1.21 |  | 6.75 | 1.24 |  | 7.06 | 1.23 |
| 20 | 45M_SP_O | M | A | Su |  | 7.72 | 1.35 |  | 6.90 | 1.23 |  | 7.06 | 1.39 |  | 7.34 | 1.00 |  | 7.26 | 1.28 |
| 21 | 09F_NE_C | F | W | Ne |  | 6.88 | 1.93 |  | 6.83 | 1.71 |  | 7.03 | 1.68 |  | 6.72 | 1.11 |  | 6.87 | 1.62 |
| 22 | 20M_NE_C | M | W | Ne |  | 6.94 | 2.24 |  | 5.90 | 2.35 |  | 6.11 | 2.05 |  | 6.41 | 1.66 |  | 6.34 | 2.10 |
| 23 | 19F_NE_C | F | A | Ne |  | 7.06 | 2.26 |  | 6.45 | 1.92 |  | 6.67 | 1.94 |  | 6.00 | 2.09 |  | 6.55 | 2.07 |
| 24 | 45M_NE_C | M | A | Ne |  | 6.97 | 1.80 |  | 6.28 | 1.71 |  | 5.86 | 2.21 |  | 5.81 | 1.79 |  | 6.22 | 1.94 |
| *Note.* NimStim = the NimStim set of facial expressions (Tottenham et al., 2009).  ^†^For the Sex, F = Female; M =Male; ^∵^For the Ethnicity, W = typical Western Caucasians; A = typical East Asians; ^♥^For the Emotion, Ha = Happiness; An = Anger; Sa = Sadness; Fe = Fear; Su = Surprise; Ne = Neutral.  ^*^The Emotional Intensity for Happiness, Anger, Sadness, Fear, and Surprise were straightforward, and for Neutral was the intensity of Peacefulness. | | | | | | | | | | | | | | | | | | | |

| Supplementary Table 1.2 | | | | | | | | | | | | | | | | | | | |
| --- | --- | --- | --- | --- | --- | --- | --- | --- | --- | --- | --- | --- | --- | --- | --- | --- | --- | --- | --- |
| *Emotional Intensity for Documentary Stimuli Based on the Current Participants* | | | | | | | | | | | | | | | | | | | |
| Stimulus information | | | | |  | Emotional Intensity^*^ | | | | | | | | | | | | | |
| No. | Resource | Sex^†^ | Ethnicity^∵^ | Emotion^♥^ |  | Australian female  (*n* = 32) | |  | Australian male  (*n* = 29) | |  | Chinese female  (*n* = 36) | |  | Chinese male  (*n* = 32) | |  | Overall  (*n* = 129) | |
|  |  |  |  |  |  | *M* | *SD* |  | *M* | *SD* |  | *M* | *SD* |  | *M* | *SD* |  | *M* | *SD* |
| 1 | Neumann et al. 2013 | F | W | Ha |  | 8.72 | 0.58 |  | 8.41 | 0.87 |  | 8.22 | 0.80 |  | 8.28 | 1.02 |  | 8.40 | 0.84 |
| 2 | Neumann et al. 2013 | M | W | Ha |  | 7.94 | 1.16 |  | 7.48 | 1.21 |  | 7.19 | 1.41 |  | 6.88 | 1.36 |  | 7.36 | 1.34 |
| 3 | Internet | F | A | Ha |  | 8.72 | 0.68 |  | 8.10 | 1.32 |  | 7.81 | 1.55 |  | 7.41 | 1.34 |  | 8.00 | 1.35 |
| 4 | Internet | M | A | Ha |  | 8.78 | 0.55 |  | 8.14 | 1.06 |  | 8.03 | 0.84 |  | 7.53 | 1.02 |  | 8.12 | 0.98 |
| 5 | Internet | F | W | An |  | 8.28 | 0.99 |  | 7.69 | 1.26 |  | 7.61 | 1.27 |  | 6.88 | 1.07 |  | 7.61 | 1.25 |
| 6 | Internet | M | W | An |  | 8.44 | 0.76 |  | 7.69 | 1.04 |  | 7.58 | 1.38 |  | 7.53 | 1.37 |  | 7.81 | 1.22 |
| 7 | Internet | F | A | An |  | 7.94 | 1.29 |  | 7.48 | 1.45 |  | 7.69 | 1.06 |  | 6.97 | 1.67 |  | 7.53 | 1.41 |
| 8 | Internet | M | A | An |  | 8.47 | 0.76 |  | 7.76 | 1.27 |  | 7.53 | 1.40 |  | 7.75 | 1.30 |  | 7.87 | 1.25 |
| 9 | Internet | F | W | Su |  | 7.47 | 1.59 |  | 6.83 | 1.69 |  | 6.47 | 1.90 |  | 6.44 | 1.81 |  | 6.79 | 1.79 |
| 10 | IAPS#4598 | M | W | Su |  | 8.72 | 0.68 |  | 8.07 | 1.16 |  | 8.03 | 1.23 |  | 8.03 | 1.06 |  | 8.21 | 1.09 |
| 11 | Internet | F | A | Su |  | 8.81 | 0.47 |  | 8.38 | 1.37 |  | 8.44 | 0.73 |  | 8.28 | 0.68 |  | 8.48 | 0.88 |
| 12 | Internet | M | A | Su |  | 8.53 | 0.57 |  | 7.83 | 1.63 |  | 7.42 | 1.61 |  | 7.69 | 1.20 |  | 7.85 | 1.38 |
| 13 | IAPS#6313 | F | W | Fe |  | 8.69 | 0.59 |  | 8.31 | 1.23 |  | 8.58 | 0.60 |  | 8.16 | 1.30 |  | 8.44 | 0.98 |
| 14 | Internet | M | W | Fe |  | 7.97 | 1.18 |  | 7.52 | 1.27 |  | 6.94 | 1.62 |  | 7.09 | 1.38 |  | 7.36 | 1.42 |
| 15 | Internet | F | A | Fe |  | 8.69 | 0.64 |  | 8.17 | 1.07 |  | 8.00 | 1.12 |  | 7.88 | 0.98 |  | 8.18 | 1.01 |
| 16 | Internet | M | A | Fe |  | 7.88 | 1.52 |  | 7.69 | 1.49 |  | 7.36 | 1.46 |  | 7.31 | 1.82 |  | 7.55 | 1.58 |
| 17 | Internet | F | W | Su |  | 7.59 | 1.32 |  | 7.21 | 1.24 |  | 7.11 | 1.47 |  | 6.69 | 1.20 |  | 7.15 | 1.34 |
| 18 | Internet | M | W | Su |  | 8.09 | 1.17 |  | 7.79 | 1.59 |  | 7.97 | 1.16 |  | 7.78 | 1.34 |  | 7.91 | 1.31 |
| 19 | Internet | F | A | Su |  | 7.91 | 0.96 |  | 7.21 | 1.15 |  | 7.17 | 1.18 |  | 7.03 | 1.18 |  | 7.33 | 1.16 |
| 20 | Internet | M | A | Su |  | 7.88 | 1.45 |  | 7.52 | 1.35 |  | 7.39 | 1.48 |  | 7.25 | 1.65 |  | 7.50 | 1.49 |
| 21 | IAPS#2377 | F | W | Ne |  | 7.31 | 1.75 |  | 6.90 | 1.70 |  | 7.00 | 1.67 |  | 6.66 | 1.33 |  | 6.97 | 1.62 |
| 22 | IAPS#2102 | M | W | Ne |  | 6.78 | 2.20 |  | 6.31 | 2.27 |  | 6.64 | 1.53 |  | 5.62 | 1.74 |  | 6.35 | 1.97 |
| 23 | Internet | F | A | Ne |  | 7.84 | 1.61 |  | 6.28 | 2.10 |  | 7.19 | 1.45 |  | 6.94 | 1.41 |  | 7.09 | 1.72 |
| 24 | Internet | M | A | Ne |  | 6.63 | 2.32 |  | 6.48 | 1.84 |  | 6.39 | 1.98 |  | 5.94 | 1.58 |  | 6.36 | 1.95 |
| *Note.* IAPS = International Affective Picture System (Lang et al., 1999). The numbers that followed the IAPS were the IAPS code for each stimulus.  ^†^For the Sex, F = Female; M =Male; ^∵^For the Ethnicity, W = typical Western Caucasians; A = typical East Asians; ^♥^For the Emotion, Ha = Happiness; An = Anger; Sa = Sadness; Fe = Fear; Su = Surprise; Ne = Neutral.  ^*^The Emotional Intensity for Happiness, Anger, Sadness, Fear, and Surprise were straightforward, and for Neutral was the intensity of Peacefulness. | | | | | | | | | | | | | | | | | | | |

| Supplementary Table 1.3 | | | | | | | | | | | | | | | | | | | |
| --- | --- | --- | --- | --- | --- | --- | --- | --- | --- | --- | --- | --- | --- | --- | --- | --- | --- | --- | --- |
| *Emotional Valence for NimStim Stimuli Based on the Current Participants* | | | | | | | | | | | | | | | | | | | |
| Stimulus information | | | | |  | Emotional Valence | | | | | | | | | | | | | |
| No. | NimStim Code | Sex^†^ | Ethnicity^∵^ | Emotion^♥^ |  | Australian female  (*n* = 32) | |  | Australian male  (*n* = 29) | |  | Chinese female  (*n* = 36) | |  | Chinese male  (*n* = 32) | |  | Overall  (*n* = 129) | |
|  |  |  |  |  |  | *M* | *SD* |  | *M* | *SD* |  | *M* | *SD* |  | *M* | *SD* |  | *M* | *SD* |
| 1 | 09F_HA_O | F | W | Ha |  | 8.53 | 0.72 |  | 7.86 | 0.92 |  | 7.44 | 0.97 |  | 7.41 | 1.01 |  | 7.80 | 1.01 |
| 2 | 20M_HA_O | M | W | Ha |  | 7.28 | 1.53 |  | 7.14 | 1.06 |  | 6.92 | 1.34 |  | 7.06 | 0.88 |  | 7.09 | 1.23 |
| 3 | 19F_HA_O | F | A | Ha |  | 8.41 | 0.76 |  | 7.14 | 1.62 |  | 6.97 | 1.32 |  | 6.59 | 1.62 |  | 7.27 | 1.51 |
| 4 | 45M_HA_O | M | A | Ha |  | 8.22 | 1.50 |  | 7.48 | 1.09 |  | 7.00 | 1.37 |  | 6.72 | 1.97 |  | 7.34 | 1.61 |
| 5 | 09F_AN_O | F | W | An |  | 2.03 | 1.12 |  | 3.17 | 1.69 |  | 2.67 | 1.31 |  | 2.13 | 0.75 |  | 2.49 | 1.32 |
| 6 | 20M_AN_O | M | W | An |  | 1.78 | 1.18 |  | 2.62 | 1.29 |  | 2.39 | 1.13 |  | 2.31 | 1.06 |  | 2.27 | 1.19 |
| 7 | 19F_AN_O | F | A | An |  | 1.75 | 1.65 |  | 2.79 | 2.09 |  | 2.17 | 1.48 |  | 2.25 | 1.67 |  | 2.22 | 1.74 |
| 8 | 45M_AN_O | M | A | An |  | 1.91 | 1.20 |  | 2.55 | 1.43 |  | 2.11 | 1.01 |  | 2.59 | 1.13 |  | 2.28 | 1.21 |
| 9 | 09F_SA_C | F | W | Sa |  | 3.06 | 1.54 |  | 3.48 | 1.24 |  | 3.11 | 1.12 |  | 3.47 | 0.88 |  | 3.27 | 1.22 |
| 10 | 20M_SA_C | M | W | Sa |  | 2.59 | 1.36 |  | 3.10 | 1.29 |  | 3.08 | 1.13 |  | 3.41 | 1.21 |  | 3.05 | 1.27 |
| 11 | 19F_SA_C | F | A | Sa |  | 2.81 | 1.28 |  | 3.38 | 1.59 |  | 3.25 | 1.02 |  | 3.63 | 1.01 |  | 3.26 | 1.25 |
| 12 | 45M_SA_C | M | A | Sa |  | 3.03 | 1.40 |  | 3.62 | 1.08 |  | 3.47 | 1.11 |  | 3.72 | 1.11 |  | 3.46 | 1.20 |
| 13 | 09F_FE_O | F | W | Fe |  | 3.75 | 1.44 |  | 4.10 | 1.50 |  | 3.58 | 1.30 |  | 3.72 | 1.28 |  | 3.78 | 1.37 |
| 14 | 20M_FE_O | M | W | Fe |  | 3.72 | 1.17 |  | 3.97 | 1.15 |  | 4.03 | 1.28 |  | 4.06 | 1.19 |  | 3.95 | 1.19 |
| 15 | 19F_FE_O | F | A | Fe |  | 3.25 | 1.34 |  | 3.69 | 1.28 |  | 3.00 | 1.49 |  | 3.00 | 1.24 |  | 3.22 | 1.36 |
| 16 | 45M_FE_O | M | A | Fe |  | 2.75 | 1.61 |  | 3.66 | 1.99 |  | 2.42 | 1.36 |  | 2.69 | 1.31 |  | 2.84 | 1.62 |
| 17 | 09F_SP_O | F | W | Su |  | 4.12 | 1.58 |  | 4.79 | 1.35 |  | 4.92 | 1.27 |  | 4.56 | 1.27 |  | 4.60 | 1.39 |
| 18 | 20M_SP_O | M | W | Su |  | 6.84 | 1.46 |  | 6.59 | 1.27 |  | 6.50 | 0.97 |  | 6.69 | 1.06 |  | 6.65 | 1.19 |
| 19 | 19F_SP_O | F | A | Su |  | 3.84 | 1.97 |  | 4.83 | 1.31 |  | 3.89 | 1.51 |  | 4.38 | 1.16 |  | 4.21 | 1.55 |
| 20 | 45M_SP_O | M | A | Su |  | 5.38 | 1.66 |  | 6.21 | 1.42 |  | 4.89 | 1.58 |  | 5.44 | 1.11 |  | 5.44 | 1.52 |
| 21 | 09F_NE_C | F | W | Ne |  | 5.00 | 0.72 |  | 5.10 | 0.94 |  | 5.08 | 0.65 |  | 5.28 | 0.58 |  | 5.12 | 0.72 |
| 22 | 20M_NE_C | M | W | Ne |  | 4.44 | 1.48 |  | 4.28 | 0.96 |  | 4.31 | 1.19 |  | 4.41 | 1.04 |  | 4.36 | 1.18 |
| 23 | 19F_NE_C | F | A | Ne |  | 4.66 | 1.04 |  | 4.97 | 0.87 |  | 4.75 | 1.08 |  | 4.75 | 0.57 |  | 4.78 | 0.91 |
| 24 | 45M_NE_C | M | A | Ne |  | 4.31 | 1.15 |  | 4.31 | 0.89 |  | 4.06 | 1.41 |  | 4.25 | 0.92 |  | 4.22 | 1.12 |
| *Note.* NimStim = the NimStim set of facial expressions (Tottenham et al., 2009).  ^†^For the Sex, F = Female; M =Male; ^∵^For the Ethnicity, W = typical Western Caucasians; A = typical East Asians; ^♥^For the Emotion, Ha = Happiness; An = Anger; Sa = Sadness; Fe = Fear; Su = Surprise; Ne = Neutral. | | | | | | | | | | | | | | | | | | | |

| Supplementary Table 1.4 | | | | | | | | | | | | | | | | | | | |
| --- | --- | --- | --- | --- | --- | --- | --- | --- | --- | --- | --- | --- | --- | --- | --- | --- | --- | --- | --- |
| *Emotional Valence for Documentary Stimuli Based on the Current Participants* | | | | | | | | | | | | | | | | | | | |
| Stimulus information | | | | |  | Emotional Valence | | | | | | | | | | | | | |
| No. | Resource | Sex^†^ | Ethnicity^∵^ | Emotion^♥^ |  | Australian female  (*n* = 32) | |  | Australian male  (*n* = 29) | |  | Chinese female  (*n* = 36) | |  | Chinese male  (*n* = 32) | |  | Overall  (*n* = 129) | |
|  |  |  |  |  |  | *M* | *SD* |  | *M* | *SD* |  | *M* | *SD* |  | *M* | *SD* |  | *M* | *SD* |
| 1 | Neumann et al. 2013 | F | W | Ha |  | 8.78 | 0.55 |  | 8.52 | 1.06 |  | 8.25 | 0.84 |  | 8.03 | 1.56 |  | 8.39 | 1.08 |
| 2 | Neumann et al. 2013 | M | W | Ha |  | 8.19 | 1.06 |  | 7.76 | 1.09 |  | 7.39 | 1.40 |  | 7.25 | 1.39 |  | 7.64 | 1.29 |
| 3 | Internet | F | A | Ha |  | 8.81 | 0.59 |  | 8.17 | 1.14 |  | 7.97 | 1.00 |  | 7.34 | 1.43 |  | 8.07 | 1.19 |
| 4 | Internet | M | A | Ha |  | 8.53 | 1.46 |  | 8.48 | 0.87 |  | 8.08 | 0.87 |  | 7.47 | 1.32 |  | 8.13 | 1.22 |
| 5 | Internet | F | W | An |  | 3.41 | 2.09 |  | 3.69 | 1.39 |  | 3.67 | 1.53 |  | 3.81 | 1.31 |  | 3.64 | 1.60 |
| 6 | Internet | M | W | An |  | 1.88 | 1.07 |  | 2.72 | 1.39 |  | 2.56 | 1.03 |  | 2.97 | 1.60 |  | 2.53 | 1.33 |
| 7 | Internet | F | A | An |  | 3.28 | 2.26 |  | 3.93 | 1.96 |  | 2.75 | 1.16 |  | 3.41 | 1.29 |  | 3.31 | 1.74 |
| 8 | Internet | M | A | An |  | 2.37 | 1.66 |  | 3.00 | 1.77 |  | 2.36 | 0.93 |  | 2.44 | 1.44 |  | 2.53 | 1.47 |
| 9 | Internet | F | W | Su |  | 1.78 | 0.97 |  | 2.45 | 1.53 |  | 3.33 | 1.39 |  | 3.13 | 1.04 |  | 2.70 | 1.38 |
| 10 | IAPS#4598 | M | W | Su |  | 4.63 | 3.53 |  | 5.34 | 2.50 |  | 3.89 | 1.92 |  | 4.72 | 2.73 |  | 4.60 | 2.73 |
| 11 | Internet | F | A | Su |  | 1.22 | 0.49 |  | 1.59 | 0.78 |  | 2.19 | 1.43 |  | 2.03 | 0.82 |  | 1.78 | 1.03 |
| 12 | Internet | M | A | Su |  | 2.69 | 1.84 |  | 3.48 | 2.13 |  | 3.86 | 1.88 |  | 3.88 | 1.93 |  | 3.49 | 1.98 |
| 13 | IAPS#6313 | F | W | Fe |  | 1.19 | 0.54 |  | 1.90 | 1.72 |  | 1.97 | 1.87 |  | 1.87 | 0.98 |  | 1.74 | 1.42 |
| 14 | Internet | M | W | Fe |  | 1.59 | 0.87 |  | 2.62 | 1.95 |  | 2.50 | 1.16 |  | 2.50 | 1.05 |  | 2.30 | 1.35 |
| 15 | Internet | F | A | Fe |  | 1.41 | 1.13 |  | 1.59 | 1.05 |  | 2.03 | 1.03 |  | 2.16 | 1.27 |  | 1.81 | 1.15 |
| 16 | Internet | M | A | Fe |  | 1.47 | 1.19 |  | 1.76 | 1.21 |  | 2.28 | 1.54 |  | 2.19 | 1.12 |  | 1.94 | 1.32 |
| 17 | Internet | F | W | Su |  | 7.59 | 1.34 |  | 7.34 | 1.11 |  | 6.50 | 1.38 |  | 6.31 | 1.55 |  | 6.91 | 1.45 |
| 18 | Internet | M | W | Su |  | 7.91 | 1.25 |  | 7.59 | 1.21 |  | 7.28 | 1.30 |  | 7.09 | 1.65 |  | 7.46 | 1.39 |
| 19 | Internet | F | A | Su |  | 8.38 | 0.79 |  | 7.17 | 1.34 |  | 7.17 | 0.97 |  | 6.84 | 1.32 |  | 7.39 | 1.25 |
| 20 | Internet | M | A | Su |  | 7.91 | 1.33 |  | 7.62 | 1.35 |  | 6.75 | 1.32 |  | 6.94 | 1.52 |  | 7.28 | 1.45 |
| 21 | IAPS#2377 | F | W | Ne |  | 5.53 | 1.32 |  | 5.38 | 1.21 |  | 5.92 | 1.25 |  | 5.69 | 0.82 |  | 5.64 | 1.17 |
| 22 | IAPS#2102 | M | W | Ne |  | 5.34 | 1.33 |  | 5.17 | 1.23 |  | 5.58 | 1.18 |  | 5.50 | 0.92 |  | 5.41 | 1.17 |
| 23 | Internet | F | A | Ne |  | 6.41 | 1.52 |  | 5.86 | 1.25 |  | 6.36 | 1.25 |  | 6.06 | 1.13 |  | 6.19 | 1.30 |
| 24 | Internet | M | A | Ne |  | 6.59 | 1.56 |  | 5.83 | 1.17 |  | 6.47 | 1.40 |  | 6.03 | 1.31 |  | 6.25 | 1.39 |
| *Note.* IAPS = International Affective Picture System (Lang et al., 1999). The numbers that followed the IAPS were the IAPS code for each stimulus.  ^†^For the Sex, F = Female; M =Male; ^∵^For the Ethnicity, W = typical Western Caucasians; A = typical East Asians; ^♥^For the Emotion, Ha = Happiness; An = Anger; Sa = Sadness; Fe = Fear; Su = Surprise; Ne = Neutral. | | | | | | | | | | | | | | | | | | | |

| Supplementary Table 1.5 | | | | | | | | | | | | | | | | | | | |
| --- | --- | --- | --- | --- | --- | --- | --- | --- | --- | --- | --- | --- | --- | --- | --- | --- | --- | --- | --- |
| *Emotional Arousal for NimStim Stimuli Based on the Current Participants* | | | | | | | | | | | | | | | | | | | |
| Stimulus information | | | | |  | Emotional Arousal | | | | | | | | | | | | | |
| No. | NimStim Code | Sex^†^ | Ethnicity^∵^ | Emotion^♥^ |  | Australian female  (*n* = 32) | |  | Australian male  (*n* = 29) | |  | Chinese female  (*n* = 36) | |  | Chinese male  (*n* = 32) | |  | Overall  (*n* = 129) | |
|  |  |  |  |  |  | *M* | *SD* |  | *M* | *SD* |  | *M* | *SD* |  | *M* | *SD* |  | *M* | *SD* |
| 1 | 09F_HA_O | F | W | Ha |  | 3.34 | 2.56 |  | 3.21 | 2.23 |  | 3.86 | 2.45 |  | 4.31 | 2.21 |  | 3.70 | 2.38 |
| 2 | 20M_HA_O | M | W | Ha |  | 2.81 | 2.43 |  | 3.28 | 1.96 |  | 3.72 | 2.48 |  | 3.41 | 2.01 |  | 3.32 | 2.25 |
| 3 | 19F_HA_O | F | A | Ha |  | 2.78 | 2.45 |  | 2.72 | 2.03 |  | 3.81 | 2.29 |  | 4.16 | 1.97 |  | 3.40 | 2.26 |
| 4 | 45M_HA_O | M | A | Ha |  | 3.53 | 2.51 |  | 4.14 | 2.34 |  | 5.11 | 2.33 |  | 4.50 | 2.08 |  | 4.35 | 2.37 |
| 5 | 09F_AN_O | F | W | An |  | 5.56 | 2.41 |  | 5.38 | 2.11 |  | 6.39 | 1.78 |  | 6.13 | 1.98 |  | 5.89 | 2.09 |
| 6 | 20M_AN_O | M | W | An |  | 5.66 | 2.39 |  | 5.83 | 2.49 |  | 6.75 | 1.59 |  | 6.53 | 1.74 |  | 6.22 | 2.10 |
| 7 | 19F_AN_O | F | A | An |  | 5.97 | 2.12 |  | 5.97 | 2.41 |  | 7.39 | 1.27 |  | 7.28 | 2.04 |  | 6.69 | 2.07 |
| 8 | 45M_AN_O | M | A | An |  | 5.59 | 2.26 |  | 5.24 | 2.54 |  | 6.69 | 1.89 |  | 6.56 | 1.81 |  | 6.06 | 2.19 |
| 9 | 09F_SA_C | F | W | Sa |  | 4.19 | 2.02 |  | 4.34 | 2.38 |  | 5.44 | 1.90 |  | 4.84 | 1.76 |  | 4.74 | 2.06 |
| 10 | 20M_SA_C | M | W | Sa |  | 4.81 | 2.22 |  | 4.10 | 2.14 |  | 5.50 | 1.92 |  | 4.72 | 1.85 |  | 4.82 | 2.07 |
| 11 | 19F_SA_C | F | A | Sa |  | 4.50 | 2.13 |  | 4.41 | 2.06 |  | 5.28 | 1.67 |  | 5.28 | 1.78 |  | 4.89 | 1.93 |
| 12 | 45M_SA_C | M | A | Sa |  | 5.03 | 2.55 |  | 4.76 | 2.18 |  | 5.69 | 2.07 |  | 4.41 | 1.95 |  | 5.00 | 2.22 |
| 13 | 09F_FE_O | F | W | Fe |  | 4.87 | 2.00 |  | 5.17 | 2.14 |  | 5.92 | 1.87 |  | 5.41 | 1.79 |  | 5.36 | 1.96 |
| 14 | 20M_FE_O | M | W | Fe |  | 4.81 | 2.07 |  | 4.69 | 2.14 |  | 5.86 | 2.00 |  | 5.19 | 2.04 |  | 5.17 | 2.09 |
| 15 | 19F_FE_O | F | A | Fe |  | 5.22 | 2.18 |  | 5.28 | 2.22 |  | 6.89 | 1.49 |  | 6.53 | 2.21 |  | 6.02 | 2.14 |
| 16 | 45M_FE_O | M | A | Fe |  | 5.00 | 2.14 |  | 4.93 | 2.30 |  | 7.17 | 1.56 |  | 6.59 | 1.74 |  | 5.98 | 2.15 |
| 17 | 09F_SP_O | F | W | Su |  | 4.63 | 2.21 |  | 4.55 | 1.86 |  | 5.86 | 1.48 |  | 5.28 | 1.95 |  | 5.12 | 1.94 |
| 18 | 20M_SP_O | M | W | Su |  | 3.75 | 2.36 |  | 4.14 | 2.15 |  | 4.25 | 1.92 |  | 3.19 | 1.71 |  | 3.84 | 2.06 |
| 19 | 19F_SP_O | F | A | Su |  | 5.09 | 2.33 |  | 4.97 | 2.32 |  | 5.94 | 1.97 |  | 5.38 | 1.93 |  | 5.37 | 2.15 |
| 20 | 45M_SP_O | M | A | Su |  | 4.44 | 2.20 |  | 4.17 | 1.98 |  | 5.83 | 1.78 |  | 5.25 | 1.97 |  | 4.97 | 2.07 |
| 21 | 09F_NE_C | F | W | Ne |  | 2.72 | 1.69 |  | 3.00 | 1.79 |  | 2.97 | 1.58 |  | 2.88 | 1.64 |  | 2.89 | 1.65 |
| 22 | 20M_NE_C | M | W | Ne |  | 3.63 | 2.01 |  | 3.86 | 2.20 |  | 4.22 | 1.64 |  | 3.22 | 1.79 |  | 3.74 | 1.92 |
| 23 | 19F_NE_C | F | A | Ne |  | 2.97 | 2.26 |  | 3.62 | 2.08 |  | 3.78 | 1.88 |  | 2.91 | 1.44 |  | 3.33 | 1.95 |
| 24 | 45M_NE_C | M | A | Ne |  | 3.87 | 2.14 |  | 3.52 | 1.60 |  | 4.42 | 1.81 |  | 3.31 | 1.77 |  | 3.81 | 1.87 |
| *Note.* NimStim = the NimStim set of facial expressions (Tottenham et al., 2009).  ^†^For the Sex, F = Female; M =Male; ^∵^For the Ethnicity, W = typical Western Caucasians; A = typical East Asians; ^♥^For the Emotion, Ha = Happiness; An = Anger; Sa = Sadness; Fe = Fear; Su = Surprise; Ne = Neutral. | | | | | | | | | | | | | | | | | | | |

| Supplementary Table 1.6 | | | | | | | | | | | | | | | | | | | |
| --- | --- | --- | --- | --- | --- | --- | --- | --- | --- | --- | --- | --- | --- | --- | --- | --- | --- | --- | --- |
| *Emotional Arousal for Documentary Stimuli Based on the Current Participants* | | | | | | | | | | | | | | | | | | | |
| Stimulus information | | | | |  | Emotional Arousal | | | | | | | | | | | | | |
| No. | Resource | Sex^†^ | Ethnicity^∵^ | Emotion^♥^ |  | Australian female  (*n* = 32) | |  | Australian male  (*n* = 29) | |  | Chinese female  (*n* = 36) | |  | Chinese male  (*n* = 32) | |  | Overall  (*n* = 129) | |
|  |  |  |  |  |  | *M* | *SD* |  | *M* | *SD* |  | *M* | *SD* |  | *M* | *SD* |  | *M* | *SD* |
| 1 | Neumann et al. 2013 | F | W | Ha |  | 3.06 | 2.49 |  | 3.34 | 2.36 |  | 5.36 | 2.53 |  | 4.53 | 2.53 |  | 4.13 | 2.63 |
| 2 | Neumann et al. 2013 | M | W | Ha |  | 3.44 | 2.47 |  | 3.90 | 2.40 |  | 4.36 | 2.09 |  | 4.25 | 1.83 |  | 4.00 | 2.21 |
| 3 | Internet | F | A | Ha |  | 3.41 | 2.84 |  | 3.45 | 2.35 |  | 4.67 | 1.91 |  | 4.13 | 2.17 |  | 3.95 | 2.36 |
| 4 | Internet | M | A | Ha |  | 3.97 | 2.92 |  | 3.66 | 2.41 |  | 5.50 | 2.10 |  | 4.50 | 2.02 |  | 4.46 | 2.46 |
| 5 | Internet | F | W | An |  | 6.06 | 2.41 |  | 5.24 | 2.42 |  | 6.06 | 1.69 |  | 6.00 | 1.72 |  | 5.86 | 2.07 |
| 6 | Internet | M | W | An |  | 5.34 | 2.38 |  | 4.86 | 2.33 |  | 6.33 | 1.82 |  | 6.03 | 1.56 |  | 5.68 | 2.09 |
| 7 | Internet | F | A | An |  | 5.66 | 2.22 |  | 5.00 | 2.24 |  | 6.19 | 1.49 |  | 6.16 | 1.63 |  | 5.78 | 1.94 |
| 8 | Internet | M | A | An |  | 5.62 | 2.59 |  | 5.31 | 2.47 |  | 6.42 | 1.25 |  | 5.72 | 1.44 |  | 5.80 | 2.01 |
| 9 | Internet | F | W | Su |  | 5.84 | 2.49 |  | 4.83 | 2.38 |  | 6.25 | 1.57 |  | 5.44 | 1.48 |  | 5.63 | 2.05 |
| 10 | IAPS#4598 | M | W | Su |  | 6.25 | 2.38 |  | 4.69 | 2.45 |  | 6.22 | 2.06 |  | 6.22 | 1.83 |  | 5.88 | 2.25 |
| 11 | Internet | F | A | Su |  | 6.47 | 2.21 |  | 6.07 | 2.63 |  | 7.25 | 1.11 |  | 6.91 | 2.04 |  | 6.71 | 2.06 |
| 12 | Internet | M | A | Su |  | 6.09 | 2.31 |  | 4.69 | 2.59 |  | 6.56 | 1.65 |  | 6.44 | 1.83 |  | 5.99 | 2.20 |
| 13 | IAPS#6313 | F | W | Fe |  | 7.34 | 1.75 |  | 5.83 | 2.71 |  | 8.17 | 0.91 |  | 7.62 | 1.68 |  | 7.30 | 2.00 |
| 14 | Internet | M | W | Fe |  | 7.13 | 1.45 |  | 5.66 | 2.51 |  | 7.22 | 1.48 |  | 6.44 | 1.98 |  | 6.65 | 1.96 |
| 15 | Internet | F | A | Fe |  | 7.25 | 2.08 |  | 6.21 | 2.64 |  | 7.81 | 1.28 |  | 7.19 | 1.69 |  | 7.16 | 2.01 |
| 16 | Internet | M | A | Fe |  | 7.22 | 1.72 |  | 5.93 | 2.74 |  | 7.33 | 1.29 |  | 7.00 | 1.59 |  | 6.91 | 1.93 |
| 17 | Internet | F | W | Su |  | 3.81 | 2.28 |  | 3.62 | 1.97 |  | 4.83 | 2.16 |  | 4.38 | 2.18 |  | 4.19 | 2.18 |
| 18 | Internet | M | W | Su |  | 3.88 | 2.32 |  | 4.38 | 2.64 |  | 5.42 | 2.03 |  | 5.19 | 2.02 |  | 4.74 | 2.31 |
| 19 | Internet | F | A | Su |  | 2.94 | 2.23 |  | 3.69 | 2.27 |  | 5.36 | 2.04 |  | 4.53 | 1.98 |  | 4.18 | 2.30 |
| 20 | Internet | M | A | Su |  | 3.75 | 2.49 |  | 4.00 | 2.35 |  | 5.08 | 2.41 |  | 5.06 | 1.87 |  | 4.50 | 2.35 |
| 21 | IAPS#2377 | F | W | Ne |  | 2.69 | 1.73 |  | 3.34 | 2.06 |  | 3.03 | 1.65 |  | 3.00 | 1.95 |  | 3.01 | 1.83 |
| 22 | IAPS#2102 | M | W | Ne |  | 2.47 | 1.52 |  | 3.03 | 1.95 |  | 3.11 | 1.80 |  | 3.25 | 1.70 |  | 2.97 | 1.75 |
| 23 | Internet | F | A | Ne |  | 2.12 | 1.81 |  | 2.66 | 2.00 |  | 2.22 | 1.48 |  | 2.34 | 1.70 |  | 2.33 | 1.73 |
| 24 | Internet | M | A | Ne |  | 1.78 | 1.54 |  | 2.34 | 1.40 |  | 2.78 | 1.84 |  | 2.66 | 1.52 |  | 2.40 | 1.62 |
| *Note.* IAPS = International Affective Picture System (Lang et al., 1999). The numbers that followed the IAPS were the IAPS code for each stimulus.  ^†^For the Sex, F = Female; M =Male; ^∵^For the Ethnicity, W = typical Western Caucasians; A = typical East Asians; ^♥^For the Emotion, Ha = Happiness; An = Anger; Sa = Sadness; Fe = Fear; Su = Surprise; Ne = Neutral. | | | | | | | | | | | | | | | | | | | |

| Supplementary Table 1.7 | | | | | | | | | | | | | | | | | | | |
| --- | --- | --- | --- | --- | --- | --- | --- | --- | --- | --- | --- | --- | --- | --- | --- | --- | --- | --- | --- |
| *Emotional Distress for NimStim Stimuli Based on the Current Participants* | | | | | | | | | | | | | | | | | | | |
| Stimulus information | | | | |  | Emotional Distress | | | | | | | | | | | | | |
| No. | NimStim Code | Sex^†^ | Ethnicity^∵^ | Emotion^♥^ |  | Australian female  (*n* = 32) | |  | Australian male  (*n* = 29) | |  | Chinese female  (*n* = 36) | |  | Chinese male  (*n* = 32) | |  | Overall  (*n* = 129) | |
|  |  |  |  |  |  | *M* | *SD* |  | *M* | *SD* |  | *M* | *SD* |  | *M* | *SD* |  | *M* | *SD* |
| 1 | 09F_HA_O | F | W | Ha |  | 1.72 | 1.22 |  | 2.07 | 1.39 |  | 2.36 | 1.57 |  | 2.38 | 1.52 |  | 2.14 | 1.45 |
| 2 | 20M_HA_O | M | W | Ha |  | 1.97 | 1.51 |  | 2.55 | 1.55 |  | 1.97 | 0.94 |  | 2.22 | 1.07 |  | 2.16 | 1.29 |
| 3 | 19F_HA_O | F | A | Ha |  | 2.25 | 2.18 |  | 2.10 | 1.21 |  | 2.53 | 1.38 |  | 2.72 | 1.37 |  | 2.41 | 1.58 |
| 4 | 45M_HA_O | M | A | Ha |  | 1.84 | 1.48 |  | 2.90 | 1.82 |  | 3.69 | 2.16 |  | 3.53 | 1.98 |  | 3.02 | 2.01 |
| 5 | 09F_AN_O | F | W | An |  | 5.06 | 2.34 |  | 4.90 | 2.40 |  | 5.78 | 1.46 |  | 5.81 | 1.73 |  | 5.41 | 2.01 |
| 6 | 20M_AN_O | M | W | An |  | 5.22 | 2.22 |  | 5.55 | 2.37 |  | 6.22 | 1.74 |  | 5.87 | 1.50 |  | 5.74 | 1.99 |
| 7 | 19F_AN_O | F | A | An |  | 5.56 | 2.31 |  | 5.69 | 2.52 |  | 6.78 | 1.71 |  | 6.75 | 1.52 |  | 6.22 | 2.09 |
| 8 | 45M_AN_O | M | A | An |  | 5.13 | 2.35 |  | 5.00 | 2.36 |  | 6.47 | 1.65 |  | 6.12 | 1.52 |  | 5.72 | 2.07 |
| 9 | 09F_SA_C | F | W | Sa |  | 4.31 | 2.29 |  | 4.48 | 2.31 |  | 5.75 | 1.70 |  | 5.50 | 1.76 |  | 5.05 | 2.09 |
| 10 | 20M_SA_C | M | W | Sa |  | 4.50 | 2.14 |  | 4.28 | 2.07 |  | 5.14 | 1.78 |  | 5.13 | 1.68 |  | 4.78 | 1.93 |
| 11 | 19F_SA_C | F | A | Sa |  | 4.63 | 2.39 |  | 4.62 | 2.35 |  | 5.58 | 1.54 |  | 5.16 | 1.80 |  | 5.02 | 2.05 |
| 12 | 45M_SA_C | M | A | Sa |  | 5.09 | 2.75 |  | 4.59 | 2.15 |  | 5.47 | 1.89 |  | 4.78 | 1.77 |  | 5.01 | 2.17 |
| 13 | 09F_FE_O | F | W | Fe |  | 4.56 | 2.23 |  | 4.55 | 2.35 |  | 5.39 | 1.79 |  | 5.06 | 1.72 |  | 4.91 | 2.03 |
| 14 | 20M_FE_O | M | W | Fe |  | 4.38 | 2.37 |  | 4.62 | 2.44 |  | 5.28 | 1.58 |  | 4.53 | 1.78 |  | 4.72 | 2.06 |
| 15 | 19F_FE_O | F | A | Fe |  | 4.78 | 2.30 |  | 4.93 | 2.31 |  | 6.42 | 1.63 |  | 5.81 | 1.47 |  | 5.53 | 2.04 |
| 16 | 45M_FE_O | M | A | Fe |  | 4.81 | 2.32 |  | 4.79 | 2.34 |  | 6.56 | 1.71 |  | 6.22 | 1.64 |  | 5.64 | 2.15 |
| 17 | 09F_SP_O | F | W | Su |  | 4.19 | 2.44 |  | 4.10 | 1.95 |  | 5.06 | 1.67 |  | 4.75 | 1.57 |  | 4.55 | 1.95 |
| 18 | 20M_SP_O | M | W | Su |  | 3.00 | 1.98 |  | 3.17 | 2.04 |  | 2.81 | 1.49 |  | 2.69 | 1.35 |  | 2.91 | 1.72 |
| 19 | 19F_SP_O | F | A | Su |  | 4.34 | 2.27 |  | 4.41 | 2.10 |  | 5.58 | 1.75 |  | 4.84 | 1.67 |  | 4.83 | 1.99 |
| 20 | 45M_SP_O | M | A | Su |  | 3.75 | 2.34 |  | 3.90 | 1.82 |  | 4.92 | 1.92 |  | 4.19 | 1.28 |  | 4.22 | 1.92 |
| 21 | 09F_NE_C | F | W | Ne |  | 2.59 | 1.74 |  | 2.93 | 1.51 |  | 3.25 | 1.83 |  | 3.03 | 1.73 |  | 2.96 | 1.71 |
| 22 | 20M_NE_C | M | W | Ne |  | 3.47 | 2.17 |  | 3.79 | 2.21 |  | 4.31 | 1.89 |  | 3.63 | 1.77 |  | 3.81 | 2.01 |
| 23 | 19F_NE_C | F | A | Ne |  | 2.87 | 2.23 |  | 3.55 | 1.97 |  | 4.19 | 1.62 |  | 3.34 | 1.64 |  | 3.51 | 1.91 |
| 24 | 45M_NE_C | M | A | Ne |  | 3.41 | 2.06 |  | 3.62 | 1.40 |  | 5.08 | 2.02 |  | 4.09 | 1.99 |  | 4.09 | 1.99 |
| *Note.* NimStim = the NimStim set of facial expressions (Tottenham et al., 2009).  ^†^For the Sex, F = Female; M =Male; ^∵^For the Ethnicity, W = typical Western Caucasians; A = typical East Asians; ^♥^For the Emotion, Ha = Happiness; An = Anger; Sa = Sadness; Fe = Fear; Su = Surprise; Ne = Neutral. | | | | | | | | | | | | | | | | | | | |

| Supplementary Table 1.8 | | | | | | | | | | | | | | | | | | | | | | | | | | | | | | | | | |
| --- | --- | --- | --- | --- | --- | --- | --- | --- | --- | --- | --- | --- | --- | --- | --- | --- | --- | --- | --- | --- | --- | --- | --- | --- | --- | --- | --- | --- | --- | --- | --- | --- | --- |
| *Emotional Distress for Documentary Stimuli Based on the Current Participants* | | | | | | | | | | | | | | | | | | | | | | | | | | | | | | | | | |
| Stimulus information | | | | | | | | |  | Emotional Distress | | | | | | | | | | | | | | | | | | | | | | | |
| No. | | Resource | | Sex^†^ | | Ethnicity^∵^ | Emotion^♥^ | |  | Australian female  (*n* = 32) | | | | |  | Australian male  (*n* = 29) | | |  | Chinese female  (*n* = 36) | | |  | | Chinese male  (*n* = 32) | | | |  | Overall  (*n* = 129) | | | |
|  |  |  |  |  |  |  |  |  |  | *M* | | *SD* | | |  | *M* | *SD* | |  | *M* | | *SD* |  | | *M* | *SD* | | |  | *M* | | | *SD* |
| 1 | | Neumann et al. 2013 | | F | | W | Ha | |  | 1.66 | | 1.43 | | |  | 2.21 | 1.24 | |  | 1.97 | | 1.00 |  | | 2.06 | 1.29 | | |  | 1.97 | | | 1.24 |
| 2 | | Neumann et al. 2013 | | M | | W | Ha | |  | 2.31 | | 1.69 | | |  | 2.69 | 1.54 | |  | 2.39 | | 1.27 |  | | 2.81 | 1.40 | | |  | 2.54 | | | 1.47 |
| 3 | | Internet | | F | | A | Ha | |  | 1.63 | | 1.31 | | |  | 2.17 | 1.10 | |  | 2.81 | | 1.43 |  | | 2.50 | 1.37 | | |  | 2.29 | | | 1.38 |
| 4 | | Internet | | M | | A | Ha | |  | 1.78 | | 1.43 | | |  | 2.41 | 1.40 | |  | 2.47 | | 0.97 |  | | 2.37 | 1.26 | | |  | 2.26 | | | 1.28 |
| 5 | | Internet | | F | | W | An | |  | 4.69 | | 2.62 | | |  | 4.79 | 2.14 | |  | 5.25 | | 1.68 |  | | 5.12 | 1.56 | | |  | 4.98 | | | 2.02 |
| 6 | | Internet | | M | | W | An | |  | 5.06 | | 2.60 | | |  | 4.76 | 2.49 | |  | 5.89 | | 1.83 |  | | 5.28 | 1.82 | | |  | 5.28 | | | 2.21 |
| 7 | | Internet | | F | | A | An | |  | 4.72 | | 2.30 | | |  | 4.34 | 1.99 | |  | 5.83 | | 1.52 |  | | 5.59 | 1.48 | | |  | 5.16 | | | 1.92 |
| 8 | | Internet | | M | | A | An | |  | 5.06 | | 2.73 | | |  | 5.07 | 2.52 | |  | 5.83 | | 1.58 |  | | 5.28 | 1.57 | | |  | 5.33 | | | 2.14 |
| 9 | | Internet | | F | | W | Su | |  | 6.22 | | 2.22 | | |  | 5.17 | 2.47 | |  | 6.36 | | 1.74 |  | | 6.06 | 1.79 | | |  | 5.98 | | | 2.08 |
| 10 | | IAPS#4598 | | M | | W | Su | |  | 5.84 | | 2.40 | | |  | 4.45 | 2.43 | |  | 5.94 | | 1.97 |  | | 5.53 | 2.53 | | |  | 5.48 | | | 2.37 |
| 11 | | Internet | | F | | A | Su | |  | 7.38 | | 1.81 | | |  | 6.34 | 2.66 | |  | 7.61 | | 1.10 |  | | 7.41 | 1.41 | | |  | 7.22 | | | 1.84 |
| 12 | | Internet | | M | | A | Su | |  | 6.81 | | 2.10 | | |  | 4.69 | 2.51 | |  | 6.36 | | 1.81 |  | | 6.38 | 1.60 | | |  | 6.10 | | | 2.14 |
| 13 | | IAPS#6313 | | F | | W | Fe | |  | 7.81 | | 1.31 | | |  | 6.21 | 2.77 | |  | 7.83 | | 1.32 |  | | 7.41 | 1.74 | | |  | 7.36 | | | 1.93 |
| 14 | | Internet | | M | | W | Fe | |  | 7.31 | | 1.33 | | |  | 5.59 | 2.64 | |  | 6.67 | | 1.67 |  | | 6.19 | 1.84 | | |  | 6.47 | | | 1.98 |
| 15 | | Internet | | F | | A | Fe | |  | 7.53 | | 2.06 | | |  | 6.48 | 2.67 | |  | 7.50 | | 1.48 |  | | 6.81 | 1.94 | | |  | 7.11 | | | 2.07 |
| 16 | | Internet | | M | | A | Fe | |  | 7.62 | | 1.62 | | |  | 6.00 | 2.99 | |  | 7.14 | | 1.64 |  | | 6.94 | 1.76 | | |  | 6.95 | | | 2.10 |
| 17 | | Internet | | F | | W | Su | |  | 2.22 | | 1.45 | | |  | 2.55 | 1.24 | |  | 2.75 | | 1.38 |  | | 2.34 | 1.23 | | |  | 2.47 | | | 1.34 |
| 18 | | Internet | | M | | W | Su | |  | 2.28 | | 1.33 | | |  | 3.07 | 1.91 | |  | 3.25 | | 1.71 |  | | 3.16 | 1.19 | | |  | 2.95 | | | 1.59 |
| 19 | | Internet | | F | | A | Su | |  | 1.72 | | 1.20 | | |  | 2.41 | 1.27 | |  | 2.53 | | 1.36 |  | | 2.84 | 1.27 | | |  | 2.38 | | | 1.33 |
| 20 | | Internet | | M | | A | Su | |  | 2.50 | | 1.88 | | |  | 2.93 | 1.65 | |  | 2.64 | | 1.48 |  | | 2.94 | 1.63 | | |  | 2.74 | | | 1.65 |
| 21 | | IAPS#2377 | | F | | W | Ne | |  | 2.47 | | 1.81 | | |  | 3.38 | 2.03 | |  | 2.67 | | 1.62 |  | | 2.53 | 1.48 | | |  | 2.74 | | | 1.75 |
| 22 | | IAPS#2102 | | M | | W | Ne | |  | 2.25 | | 1.63 | | |  | 2.76 | 1.98 | |  | 2.83 | | 1.54 |  | | 2.78 | 1.70 | | |  | 2.66 | | | 1.70 |
| 23 | | Internet | | F | | A | Ne | |  | 1.84 | | 1.80 | | |  | 2.55 | 1.88 | |  | 1.89 | | 1.04 |  | | 2.09 | 1.35 | | |  | 2.08 | | | 1.54 |
| 24 | | Internet | | M | | A | Ne | |  | 1.56 | | 1.29 | | |  | 1.97 | 1.05 | |  | 1.97 | | 1.21 |  | | 2.25 | 1.44 | | |  | 1.94 | | | 1.27 |
| *Note.* IAPS = International Affective Picture System (Lang et al., 1999). The numbers that followed the IAPS were the IAPS code for each stimulus.  ^†^For the Sex, F = Female; M =Male; ^∵^For the Ethnicity, W = typical Western Caucasians; A = typical East Asians; ^♥^For the Emotion, Ha = Happiness; An = Anger; Sa = Sadness; Fe = Fear; Su = Surprise; Ne = Neutral. | | | | | | | | | | | | | | | | | | | | | | | | | | | | | | | | | |
| Supplementary Table 1.9 | | | | | | | | | | | | | | | | | | | | | | | | | | | | | | |  |  |  |
| *Stimulus Description and Empathic Accuracies (ACC) for NimStim Stimuli Based on the Current Participants* | | | | | | | | | | | | | | | | | | | | | | | | | | | | | | |  |  |  |
| Stimulus information | | | | | | | | | | | |  | ACC (%) | | | | | | | | | | | | | | | | | |  |  |  |
| No. | | NimStim Code | | Sex | | | Ethnicity | | | Emotion | |  | Australian female  (*n* = 32) | | | | Australian  male  (*n* = 29) | | | Chinese  female  (*n* = 36) | | | Chinese  male  (*n* = 32) | | | Overall  (*n* = 129) | | | | |  |  |  |
| 1 | | 09F_HA_O | | Female | | | Caucasian | | | Happiness | |  | 100.0 | | | | 96.6 | | | 100.0 | | | 100.0 | | | 99.2 | | | | |  |  |  |
| 2 | | 20M_HA_O | | Male | | | Caucasian | | | Happiness | |  | 100.0 | | | | 96.6 | | | 94.4 | | | 100.0 | | | 97.7 | | | | |  |  |  |
| 3 | | 19F_HA_O | | Female | | | Asian | | | Happiness | |  | 100.0 | | | | 100.0 | | | 97.2 | | | 96.9 | | | 98.4 | | | | |  |  |  |
| 4 | | 45M_HA_O | | Male | | | Asian | | | Happiness | |  | 93.8 | | | | 93.1 | | | 94.4 | | | 96.9 | | | 94.6 | | | | |  |  |  |
| 5 | | 09F_AN_O | | Female | | | Caucasian | | | Anger | |  | 100.0 | | | | 89.7 | | | 91.7 | | | 100.0 | | | 95.3 | | | | |  |  |  |
| 6 | | 20M_AN_O | | Male | | | Caucasian | | | Anger | |  | 93.8 | | | | 96.6 | | | 97.2 | | | 100.0 | | | 96.9 | | | | |  |  |  |
| 7 | | 19F_AN_O | | Female | | | Asian | | | Anger | |  | 100.0 | | | | 96.6 | | | 97.2 | | | 100.0 | | | 98.4 | | | | |  |  |  |
| 8 | | 45M_AN_O | | Male | | | Asian | | | Anger | |  | 100.0 | | | | 89.7 | | | 97.2 | | | 96.9 | | | 96.1 | | | | |  |  |  |
| 9 | | 09F_SA_C | | Female | | | Caucasian | | | Sadness | |  | 100.0 | | | | 96.6 | | | 86.1 | | | 71.9 | | | 88.4 | | | | |  |  |  |
| 10 | | 20M_SA_C | | Male | | | Caucasian | | | Sadness | |  | 96.9 | | | | 96.6 | | | 88.9 | | | 84.4 | | | 91.5 | | | | |  |  |  |
| 11 | | 19F_SA_C | | Female | | | Asian | | | Sadness | |  | 96.9 | | | | 96.6 | | | 88.9 | | | 93.8 | | | 93.8 | | | | |  |  |  |
| 12 | | 45M_SA_C | | Male | | | Asian | | | Sadness | |  | 59.4 | | | | 51.7 | | | 83.3 | | | 93.8 | | | 72.9 | | | | |  |  |  |
| 13 | | 09F_FE_O | | Female | | | Caucasian | | | Fear | |  | 68.8 | | | | 55.2 | | | 58.3 | | | 50.0 | | | 58.1 | | | | |  |  |  |
| 14 | | 20M_FE_O | | Male | | | Caucasian | | | Fear | |  | 56.3 | | | | 41.4 | | | 19.4 | | | 50.0 | | | 41.1 | | | | |  |  |  |
| 15 | | 19F_FE_O | | Female | | | Asian | | | Fear | |  | 62.5 | | | | 62.1 | | | 63.9 | | | 75.0 | | | 65.9 | | | | |  |  |  |
| 16 | | 45M_FE_O | | Male | | | Asian | | | Fear | |  | 84.4 | | | | 65.5 | | | 86.1 | | | 71.9 | | | 77.5 | | | | |  |  |  |
| 17 | | 09F_SP_O | | Female | | | Caucasian | | | Surprise | |  | 75.0 | | | | 82.8 | | | 91.7 | | | 87.5 | | | 84.5 | | | | |  |  |  |
| 18 | | 20M_SP_O | | Male | | | Caucasian | | | Surprise | |  | 84.4 | | | | 62.1 | | | 36.1 | | | 34.4 | | | 53.5 | | | | |  |  |  |
| 19 | | 19F_SP_O | | Female | | | Asian | | | Surprise | |  | 65.6 | | | | 75.9 | | | 72.2 | | | 90.6 | | | 76.0 | | | | |  |  |  |
| 20 | | 45M_SP_O | | Male | | | Asian | | | Surprise | |  | 90.6 | | | | 93.1 | | | 69.4 | | | 100.0 | | | 87.6 | | | | |  |  |  |
| 21 | | 09F_NE_C | | Female | | | Caucasian | | | Neutral | |  | 93.8 | | | | 96.6 | | | 88.9 | | | 100.0 | | | 94.6 | | | | |  |  |  |
| 22 | | 20M_NE_C | | Male | | | Caucasian | | | Neutral | |  | 81.2 | | | | 75.9 | | | 72.2 | | | 84.4 | | | 78.3 | | | | |  |  |  |
| 23 | | 19F_NE_C | | Female | | | Asian | | | Neutral | |  | 90.6 | | | | 89.7 | | | 94.4 | | | 93.8 | | | 92.2 | | | | |  |  |  |
| 24 | | 45M_NE_C | | Male | | | Asian | | | Neutral | |  | 71.9 | | | | 65.5 | | | 61.1 | | | 75.0 | | | 68.2 | | | | |  |  |  |
| *Note.* NimStim = the NimStim set of facial expressions (Tottenham et al., 2009). | | | | | | | | | | | | | | | | | | | | | | | | | | |  | | |  |  |  |  |

| Supplementary Table 1.10 | | | | | | | | | | | | | |
| --- | --- | --- | --- | --- | --- | --- | --- | --- | --- | --- | --- | --- | --- |
| *Stimulus Description and Empathic Accuracies (ACC) for Documentary Stimuli Based on the Current Participants* | | | | | | | | | | | | | |
| Stimulus information | | | | | |  | ACC (%) | | | | | | |
| No. | Resource | Sex | Ethnicity | Description of the background | Emotion |  | Australian female  (*n* = 32) | Australian  male  (*n* = 29) | Chinese  female  (*n* = 36) | Chinese  male  (*n* = 32) | | Overall  (*n* = 129) | |
| 1 | Neumann et al. 2013 | Female | Caucasian | a bride at her wedding party | Happiness |  | 100.0 | 100.0 | 100.0 | 100.0 | | 100.0 | |
| 2 | Neumann et al. 2013 | Male | Caucasian | a participant running a marathon | Happiness |  | 100.0 | 100.0 | 100.0 | 100.0 | | 100.0 | |
| 3 | Internet | Female | Asian | a bride at her wedding party | Happiness |  | 96.9 | 100.0 | 100.0 | 100.0 | | 99.2 | |
| 4 | Internet | Male | Asian | a participant running a marathon | Happiness |  | 100.0 | 96.6 | 100.0 | 100.0 | | 99.2 | |
| 5 | Internet | Female | Caucasian | a protester in a strike parade | Anger |  | 100.0 | 96.6 | 97.2 | 100.0 | | 98.4 | |
| 6 | Internet | Male | Caucasian | a driver shouting to others | Anger |  | 96.9 | 100.0 | 86.1 | 100.0 | | 95.3 | |
| 7 | Internet | Female | Asian | a protester in a strike parade | Anger |  | 90.6 | 93.1 | 97.2 | 100.0 | | 95.3 | |
| 8 | Internet | Male | Asian | a driver shouting to others | Anger |  | 93.8 | 96.6 | 88.9 | 96.9 | | 93.8 | |
| 9 | Internet | Female | Caucasian | a victim of a bushfire hugging her children | Sadness |  | 87.5 | 86.2 | 91.7 | 93.8 | | 89.9 | |
| 10 | IAPS#4598 | Male | Caucasian | a crying soldier hugging a lady | Sadness |  | 65.6 | 65.5 | 80.6 | 75.0 | | 72.1 | |
| 11 | Internet | Female | Asian | a victim of an earthquake hugging her child | Sadness |  | 96.9 | 96.6 | 97.2 | 100.0 | | 97.7 | |
| 12 | Internet | Male | Asian | a crying soldier hugging a lady | Sadness |  | 90.6 | 93.1 | 86.1 | 87.5 | | 89.1 | |
| 13 | IAPS#6313 | Female | Caucasian | a hostage threatened by a knife-armed robber | Fear |  | 96.9 | 100.0 | 100.0 | 96.9 | | 98.4 | |
| 14 | Internet | Male | Caucasian | a hostage threatened by a knife-armed robber | Fear |  | 96.9 | 96.6 | 94.4 | 96.9 | | 96.1 | |
| 15 | Internet | Female | Asian | a hostage threatened by a knife-armed robber | Fear |  | 100.0 | 96.6 | 94.4 | 100.0 | | 97.7 | |
| 16 | Internet | Male | Asian | a hostage threatened by a knife-armed robber | Fear |  | 90.6 | 89.7 | 86.1 | 87.5 | | 88.4 | |
| 17 | Internet | Female | Caucasian | a person holding gift boxes | Surprise |  | 93.8 | 100.0 | 88.9 | 90.6 | | 93.0 | |
| 18 | Internet | Male | Caucasian | a person reading news on a laptop | Surprise |  | 68.8 | 75.9 | 27.8 | 40.6 | | 51.9 | |
| 19 | Internet | Female | Asian | a person opening a gift box | Surprise |  | 90.6 | 82.8 | 52.8 | 56.3 | | 69.8 | |
| 20 | Internet | Male | Asian | a person reading news on a laptop | Surprise |  | 56.3 | 62.1 | 36.1 | 37.5 | | 47.3 | |
| 21 | IAPS#2377 | Female | Caucasian | a person reading a book in a courtyard | Neutral |  | 93.8 | 96.6 | 91.7 | 100.0 | | 95.3 | |
| 22 | IAPS#2102 | Male | Caucasian | a person reading a newspaper on a wooden bench | Neutral |  | 90.6 | 79.3 | 97.2 | 96.9 | | 91.5 | |
| 23 | Internet | Female | Asian | a person reading a book in front of a bookshelf | Neutral |  | 93.8 | 75.9 | 91.7 | 96.9 | | 89.9 | |
| 24 | Internet | Male | Asian | a person reading a newspaper on a sofa | Neutral |  | 71.9 | 75.9 | 77.8 | 87.5 | | 78.3 | |
| *Note.* IAPS = International Affective Picture System (Lang et al., 1999). The numbers that followed the IAPS were the IAPS code for each stimulus.  For all Documentary stimuli, there was an arrow pointed to the main character for participants to empathize with. | | | | | | | | | | |  | |  |

References

Lang, P. J., Bradley, M. M., & Cuthbert, B. N. (1999). International affective picture system (IAPS): Technical manual and affective ratings. Gainesville, FL: The Center for Research in Psychophysiology, University of Florida.

Neumann, D. L., Boyle, G. J., & Chan, R. C. (2013). Empathy towards individuals of the same and different ethnicity when depicted in negative and positive contexts. *Personality and Individual Differences, 55,* 8-13.

Tottenham, N., Tanaka, J. W., Leon, A. C., McCarry, T., Nurse, M., Hare, T. A., . . . Nelson, C. (2009). The NimStim set of facial expressions: judgments from untrained research participants. *Psychiatry Research, 168,* 242-249. doi: 10.1016/j.psychres.2008.05.006
