# Supplementary material for: Culture, Sex, and Group-Bias in Trait and State Empathy
Source: Front Psychol. 2021 Apr 28;12:561930. doi: 10.3389/fpsyg.2021.561930 (PMC8113867; doi:10.3389/fpsyg.2021.561930)
Supplement: Supplementary file 2 [file Table_2.docx]

Culture, Sex, and Group-Bias in Trait and State Empathy

**Supplementary Document 2**

**Pilot Testing and Adaptation**

Pilot testing was conducted for Task I (i.e., NimStim stimuli) and Task II (i.e., Documentary stimuli), separately. For each task, two pilot tests were conducted. In the first pilot test (*n* = 2), two participants reported that since the question list (i.e., Q1 to Q8) presented after each stimulus was long, they had trouble retaining the stimulus in memory clearly until finishing all of the questions. This issue was more significant for the stimuli of Task I (i.e., NimStim stimuli) than that of Task II (i.e., Documentary stimuli).

Therefore, the current authors presented both Tasks I and II twice (i.e., each time presenting a part of the questions from the list): In the first presentation, participants were asked to answer the empathy-related questions from the list (i.e., Q3, Q4, Q6, Q7, and Q8). In the second presentation, participants were asked to answer the cognitive judgment-related questions from the list (i.e., Q 1, Q2, and Q5). In the second pilot test (*n* = 5), five participants confirmed that instructions for the adapted tasks were clear and all of them could finish these tasks without the aforementioned or issue or others.
