# Supplementary material for: Culture, Sex, and Group-Bias in Trait and State Empathy
Source: Front Psychol. 2021 Apr 28;12:561930. doi: 10.3389/fpsyg.2021.561930 (PMC8113867; doi:10.3389/fpsyg.2021.561930)
Supplement: Supplementary file 3 [file Table_3.docx]

Culture, Sex, and Group-Bias in Trait and State Empathy

**Supplementary Document 3**

**Correlations between Trait Empathy Scores and State Empathy**

Pearson’s correlation coefficients (*r*) were calculated amongst participants’ trait empathy scores (i.e., the EQ and IRI scores), state empathy for the NimStim stimuli (i.e., the computer-based Task I), and state empathy for the Documentary stimuli (i.e., the computer-based Task II). The scales of trait empathy were scored according to the official scoring rules of each scale; namely, the EQ according to Baron-Cohen and Wheelwright (2004), and the IRI according to Davis (1980). For Task I, the total score of all empathy responses (i.e., emotional empathy and cognitive empathy) was calculated based on all NimStim stimuli. For Task II, the total score of all empathy responses (i.e., emotional empathy, cognitive empathy, perspective-taking) was calculated based on all Documentary stimuli.

Meanwhile, in the following table, the correlation results are presented separately for the Australian participants (i.e., above diagonal) and the Chinese participants (i.e., below diagonal). Since the bivariate correlation analysis is sensitive to univariate outliers (*z*-score > 3.29), distributions of all variables were examined based on the Australian participant group and Chinese participant group, separately. Any score with univariate outliers was converted (the converted variables were marked by ^†^ in the following table, and their conversion formulas were presented in the footnote of the table). After the conversion, no more univariate outliers were identified (all *z*-score < 3.29).

| Table S3.1 | | | | | | | | | | |
| --- | --- | --- | --- | --- | --- | --- | --- | --- | --- | --- |
| *Pearson’s Correlation Coefficients amongst Trait Empathy Scores, State Empathy for NimStim Stimuli (Task I), and State Empathy for Documentary Stimuli (Task II) based on the Current Australian (n = 61, above diagonal) and Chinese Participants (n = 68, below diagonal)* | | | | | | | | | | |
|  | EQ-40 | IRI-EC | IRI-PT | IRI-PD | IRI-FS^†^ | EM-I | CM-I | EM-II^†^ | CM-II^†^ | PT-II |
| EQ-40 | / | .68^***^ | .73^***^ | -.14 | .39^**^ | .01 | .09 | .18 | .18 | .45^***^ |
| IRI-EC | .45^***^ | / | .72^***^ | .06 | .49^***^ | .25 | -.06 | .41^***^ | .03 | .30^*^ |
| IRI-PT | .48^***^ | .21 | / | -.12 | .29^*^ | .15 | .02 | .25 | .03 | .33^**^ |
| IRI-PD | -.06 | .43^***^ | -.12 | / | .13 | -.10 | -.46^***^ | .11 | -.25 | -.26^*^ |
| IRI-FS^†^ | .51^***^ | .47^***^ | .18 | .34^**^ | / | .30^*^ | .08 | .55^***^ | .17 | .36^**^ |
| EM for Task I | .21 | .15 | .05 | -.23 | .12 | / | .10 | .75^***^ | -.06 | .07 |
| CM for Task I | .24 | .02 | .05 | -.19 | .07 | .60^***^ | / | -.05 | .68^***^ | .43^***^ |
| EM for Task II^†^ | .41^***^ | .13 | .21 | -.24 | .13 | .72^***^ | .42^***^ | / | .08 | .26^*^ |
| CM for Task II^†^ | .41^***^ | .14 | .21 | -.16 | .11 | .44^***^ | .49^***^ | .71^***^ | / | .50^***^ |
| PT for Task II | .38^**^ | .24^*^ | .24^*^ | -.04 | .19 | .57^***^ | .46^***^ | .69^***^ | .62^***^ | / |
| *Note.* EQ = Empathy Quotient; EQ-40 = total score for the 40-item EQ; IRI = Interpersonal Reactivity Index; IRI-PT = total score for the IRI perspective-taking items; IRI-EC = total score for the IRI empathic concern items; IRI-PD = total score for the IRI personal distress items; IRI-FS = total score for the IRI fantasy items. ‘-I’ = Task I; ‘-II’ = Task II; EM = emotional empathy (i.e., the question was “I felt _____ the feeling of the main character. 1 = *not at all* to 9 = *very strongly*”); CM = cognitive empathy (i.e., the question was “I understood _____ the situation of the main character. 1 = *not at all* to 9 = *very fully*”); PT = perspective-taking (i.e., the question was I can _____ imagine myself in the situation of the main character. 1 = *not at all* to 9 = *very easily*”).  ^†^Univariate outliers (i.e., *z*-scores > 3.29) were identified for the original scores of IRI-FS, EM-II, and CM-II. To eliminate the impacts of univariate outliers, these variables were converted properly [i.e., the conversion formula for IRI-FS was $\text{5- √(29-IRI-FS)}$, for EM-II was $1\text{5- }\text{√(}\text{2}\text{15}\text{ }\text{-}\text{ EM-II}\text{)}$, and for CM-II was $\text{10}\text{- √(}\text{216}\text{ }\text{–}\text{ CM-II}\text{)}$].  ^*^*p* < .05, ^**^*p* < .01, ^***^ *p* < .001. | | | | | | | | | | |

References

Baron-Cohen, S., & Wheelwright, S. (2004). The Empathy Quotient: An investigation of adults with Asperger syndrome or high functioning autism, and normal sex differences. *Journal of Autism and Developmental Disorders, 34,* 163-175. doi: 10.1023/B:JADD.0000022607.19833.00

Davis, M. H. (1980). A multidimensional approach to individual differences in empathy. *Journal Supplement Abstract Service Catalog of Selected Documents in Psychology, 10,* 85-85.
