# Supplementary material for: Culture, Sex, and Group-Bias in Trait and State Empathy
Source: Front Psychol. 2021 Apr 28;12:561930. doi: 10.3389/fpsyg.2021.561930 (PMC8113867; doi:10.3389/fpsyg.2021.561930)
Supplement: Supplementary file 4 [file Table_4.docx]

Culture, Sex, and Group-Bias in Trait and State Empathy

**Supplementary Document 4**

**The Full Introduction of the State Empathy Results**

### State Empathy for the NimStim Stimuli (Table 5)

Omnibus test. ***Four-way interaction.*** One four-way interaction, Participant Culture × Participant Sex × Stimulus Ethnicity × Stimulus Sex [*F* (1, 5873.99) = 5.11, *p* = .024], was significant. Pairwise comparisons yielded the following results. First, Chinese female participants showed an ethnic out-group bias on Male stimuli (i.e., Caucasian Male > Asian Male stimuli, *p* = .011, 95% CI = [0.07, 0.55]). Second, Australian male participants had the sex out-group favor (i.e., female > male stimuli) on both Caucasian stimuli (*p* = .037, 95% CI = [0.02, 0.55]) and Asian stimuli (*p* = .005, 95% CI = [0.11, 0.65]).

***Three-way interaction.*** One three-way interaction was identified, namely, Participant Culture × Stimulus Ethnicity × Stimulus Emotion^[[1]](#footnote-1)^ [*F* (5, 5874.06) = 2.59, *p* = .024]. Results of the pairwise comparisons were three-fold. First, the Participant Culture effects were significant on five stimuli, namely, Caucasian Happiness (i.e., Australian > Chinese participant, *p* = .019, 95% CI = [0.10, 1.11]), Asian Happiness (i.e., Australian > Chinese participant, *p* = .001, 95% CI = [0.33, 1.34]), Asian Neutral-Peacefulness (i.e., Australian > Chinese participant, *p* = .006, 95% CI = [0.20, 1.21]), Asian Anger (i.e., Chinese > Australian participant, *p* = .047, 95% CI = [0.01, 1.02]), and Asian Fear (i.e., Chinese > Australian participant, *p* = .001, 95% CI = [0.32, 1.33]). Second, the Stimulus Ethnicity effect on Neutral-Peacefulness was consistent (i.e., both were Caucasian > Asian stimuli) between Australian participants (*p* = .031, 95% CI = [0.03, 0.67]) and Chinese participants (*p* < .001, 95% CI = [0.36, 0.97]). Specifically, Chinese participants exhibited an ethnic in-group bias in empathy for Fear (i.e., Asian > Caucasian stimuli, *p* = .035, 95% CI = [0.02, 0.64]) and an ethnic out-group bias in empathy for Sadness (i.e., Caucasian > Asian stimuli, *p* = .004, 95% CI = [0.14, 0.75]).

***Two-way interaction.*** Six^[[2]](#footnote-2)^ significant two-way interactions were identified. (1) Empathy Task × Participant Culture [*F* (1, 5873.99) = 96.45, *p* < .001]. Results of pairwise comparisons revealed the following effects. First, the Empathy Task effects were significant for both Australian (i.e., cognitive > emotional empathy, *p* < .001, 95% CI = [0.31, 0.58]) and Chinese participants (i.e., emotional > cognitive empathy, *p* < .001, 95% CI = [0.33, 0.58]). Second, the Participant Culture effects were significant for both emotional empathy (i.e., Chinese > Australian participant, *p* = .029, 95% CI = [0.50, 0.89]) and cognitive empathy (i.e., Australian > Chinese participant, *p* = .042, 95% CI = [0.02, 0.85]).

(2) Empathy Task × Participant Sex [*F* (1, 5873.99) = 28.60, *p* < .001]. Pairwise comparisons of this interaction suggested the following two points. First, the Empathy Task effects differed between male (i.e., emotional > cognitive empathy, *p* < .001, 95% CI = [0.12, 0.38]) and female participants (i.e., cognitive > emotional empathy, *p* < .001, 95% CI = [0.12, 0.36]). Second, the Participant Sex effect was significant for cognitive empathy (i.e., female > male participant, *p* = .039, 95% CI = [0.02, 0.86]).

(3) Participant Sex × Stimuli Emotion^1^ [*F* (5, 5874.01) = 3.44, *p* = .004]. Pairwise results revealed that the Participant Sex effect was significant on Neutral-Peacefulness (i.e., female > male participants, *p* = .014, 95% CI = [0.12, 1.03]).

*Main effect.* Although two^[[3]](#footnote-3)^ main effects were significant, they were both qualified by the significant higher-order interactions.

Follow-up analyses. Since the above omnibus test revealed significant interaction effects for the Empathy Task, follow-up analyses were conducted for two types of the Empathy Task of Task I (viz., emotional empathy and cognitive empathy), separately (adjusted significance level α = .025).

*Emotional empathy*. *Two-way interaction.* Three^[[4]](#footnote-4)^ two-way interactions were identified. (1) Participant Culture × Stimulus Emotion^1^ [*F* (5, 2874.00) = 11.81, *p* < .001]. According to pairwise comparisons, the Participant Culture effect (i.e., both were Chinese > Australian participant) was significant for both Anger (*p* = .002, 95% CI = [0.35, 1.57]) and Fear (*p* = .005, 95% CI = [0.27, 1.49]).

(2) Participant Sex × Stimulus Emotion^1^ [*F* (5, 2874.01) = 4.17, *p* < .001]. However, the Participant Sex effect was no longer significant when the pairwise comparisons were made for each stimulus emotion (all *p*s ≥ .158).

*Main effect.* Two^[[5]](#footnote-5)^ main effects were significant, including (1) Stimulus Sex [i.e., female > male stimuli, *F* (1, 2874.23) = 8.61, *p* = .003].

***Cognitive empathy***. *Four-way interaction.* A marginally significant four-way interaction emerged; that is, Participant Culture × Participant Sex × Stimulus Ethnicity × Stimulus Sex [*F* (1, 2873.98) = 4.24, *p* = .040]. According to the pairwise comparisons, Australian male participants had a significant sex out-group favor on the Asian stimuli (i.e., female > male stimuli, *p* = .006, 95% CI = [0.14, 0.83]).

*Two-way interaction.* Three^[[6]](#footnote-6)^ two-way interactions were found. (1) Participant Culture × Stimulus Emotion^1^ [*F* (5, 2873.99) = 17.10, *p* < .001]. Pairwise comparisons indicated that the Participant Culture effect (i.e., both were Australian > Chinese participant; both *p*s < .001) was significant for Happiness (95% CI = [0.73, 1.76]) and Neutral-Peacefulness (95% CI = [0.60, 1.63]).

*Main effect.* Although there were two^[[7]](#footnote-7)^ significant main effects, both were qualified by the above interactions.

### State Empathy for the Documentary Stimuli (Table 5)

Omnibus test. ***Four-way interaction.*** One four-way interaction was identified, namely, Participant Culture × Participant Sex × Stimulus Sex × Stimulus Emotion^1^ [*F* (5, 8874.01) = 3.85, *p* = .002]. Results of its pairwise comparisons covered the following three aspects. First, the Participant Culture effect (i.e., all were Chinese > Australian participant) was identified with male participants on three stimuli (*p* = .024, 95% CI = [0.08, 1.18], for Female Fear; *p* = .003, 95% CI = [0.29, 1.39], for Male Surprise; *p* = .001, 95% CI = [0.35, 1.44], for Male Neutral-Peacefulness) and with female participants on three stimuli (*p* = .002, 95% CI = [0.31, 1.35], for Female Fear; *p* = .008, 95% CI = [0.19, 1.22], for Male Fear; *p* = .029, 95% CI = [0.06, 1.10], for Male Surprise).

Second, the Participant Sex effect (i.e., all were female > male participant) was identified with Australian participants on six stimuli (*p* < .001, 95% CI = [0.84, 1.94], for Female Happiness; *p* = .034, 95% CI = [0.04, 1.14], for Female Surprise; *p* = .015, 95% CI = [0.13, 1.23], for Male Sadness; *p* = .014, 95% CI = [0.14, 1.24], for Male Anger; *p* = .011, 95% CI = [0.16, 1.26], for Male Surprise; *p* < .001, 95% CI = [0.67, 1.77], for Male Neutral-Peacefulness) and also identified with Chinese participants on two stimuli (*p* = .023, 95% CI = [0.09, 1.12], for Female Happiness; *p* = .029, 95% CI = [0.06, 1.10], for Female Fear).

Third, in the aspect of the Stimulus Sex variable, the sex in-group favor was observed for overall empathy for Happiness with both Australian male participants (i.e., male > female stimuli, *p* = .029, 95% CI = [0.04, 0.75]) and Australian female participants (i.e., female > male stimuli, *p* < .001, 95% CI = [0.39, 1.06]). Meanwhile, a sex in-group favor (i.e., female > male stimuli) on overall empathy for Fear was shown by both Australian female participants (i.e., *p* = .029, 95% CI = [0.04, 0.71] and Chinese female participants (*p* = .002, 95% CI = [0.19, 0.82]). Nevertheless, Australian female participants had a sex out-group favor (i.e., male > female stimuli) on overall empathy for Sadness (*p* < .001, 95% CI = [0.24, 0.92]) and Anger (*p* = .016, 95% CI = [0.08, 0.75]). In addition, the Stimulus Sex effect (i.e., female > male stimuli) was consistent for the stimuli of Surprise and Neutral-Peacefulness amongst all culture–sex participants groups, namely, Australian male participants (both *p*s < .001; 95% CI = [0.87, 1.58], for Surprise; 95% CI = [1.37, 2.08], for Neutral-Peacefulness), Australian female participants (both *p*s < .001; 95% CI = [0.77, 1.45] for Surprise; 95% CI = [0.54, 1.22], for Neutral-Peacefulness), Chinese male participants (*p* = .013, 95% CI = [0.09, 0.77], for Surprise; *p* < .001, 95% CI = [0.65, 1.33], for Neutral-Peacefulness), and Chinese female participants (*p* = .002, 95% CI = [0.18, 0.82], for Surprise; *p* < .001, 95% CI = [0.83, 1.47], for Neutral-Peacefulness).

***Three-way interaction.*** Six^[[8]](#footnote-8)^ three-way interactions were identified. (1) Empathy Task1 × Participant Culture × Stimulus Emotion^1^ [*F* (10, 8874.00) = 4.80, *p* < .001]. Pairwise comparisons revealed that the Participant Culture effect (i.e., all were Chinese > Australian participant) was significant in four comparisons. They were emotional empathy for Surprise (i.e., *p* = .001, 95% CI = [0.27, 1.10]), emotional empathy for Neutral-Peacefulness (*p* = .017, 95% CI = [0.09, 0.92]), perspective-taking for Sadness (*p* = .021, 95% CI = [0.07, 0.90]), and perspective-taking for Fear (*p* < .001, 95% CI = [0.98, 1.80]).

(2) Participant Culture × Stimulus Ethnicity × Stimulus Emotion^1^ [*F* (5, 8874.04) = 3.24, *p* = .006]. The pairwise comparisons revealed the following results. First, the Participant Culture effect (i.e., all were Chinese > Australian participant) was identified on four types of stimuli; namely, Caucasian Fear (*p* < .001, 95% CI = [0.29, 1.04]), Asian Fear (*p* = .001, 95% CI = [0.24, 1.00]), Asian Surprise (*p* = .049, 95% CI = [< 0.01, 0.76]), and Asian Neutral-Peacefulness (*p* = .012, 95% CI = [0.11, 0.86]). Second, in light of Neutral-Peacefulness, the Stimulus Ethnicity effect (i.e., both were Asian > Caucasian stimuli) was identified with both Australian participants (*p* = .003, 95% CI = [0.13, 0.62]) and Chinese participants (*p* < .001, 95% CI = [0.49, 0.96]). Third, Australian participants had a significant ethnic out-group bias on Happiness (i.e., Asian > Caucasian stimuli, *p* = .020, 95% CI = [0.05, 0.54]). In contrast, Chinese participants had a significant ethnic in-group bias on Sadness (i.e., Asian > Caucasian stimuli, *p* < .001, 95% CI = [0.16, 0.63]).

***Two-way interaction.*** Seven^[[9]](#footnote-9)^ significant two-way interactions were identified. Nevertheless, all of them were qualified by the above higher-order interactions.

*Main effect.* Five^[[10]](#footnote-10)^ main effects were significant, with their result interpretations qualified by the above interactions.

Follow-up analyses. Since the above omnibus test revealed significant interaction effects on the Empathy Task, follow-up analyses were conducted for the three types of the Empathy Task of Task II (i.e., emotional empathy, cognitive empathy, and perspective-taking), correspondingly (adjusted significance level α = .017).

*Emotional empathy*. *Four-way interaction.* One four-way interaction was marginally significant, which was Participant Culture × Participant Sex × Stimulus Sex × Stimulus Emotion^1^ [*F* (5, 2874.01) = 2.66, *p* = .021]. The following results were indicated by the pairwise comparisons. First, the Participant Culture effect (i.e., all were Chinese > Australian participant) was significant on Male Surprise with both male and female participants (*p* =.015, 95% CI = [0.21, 1.89], for male participant; *p* =.016, 95% CI = [0.18, 1.78], for female participant), and was also significant on Male Neutral-Peacefulness with male participants (*p* =.002, 95% CI = [0.49, 2.17]). Second, the Participant Sex effect (i.e., both were female > male participant) was significant with Australian participants on both Female Happiness (*p* = .006, 95% CI = [0.34, 2.02]) and Male Neutral-Peacefulness (*p* <.001, 95% CI = [0.74, 2.42]). Third, the Stimulus Sex effect (i.e., female > male stimuli) was significant with three culture–sex participant groups, namely, Australian male participants (*p* = .003, 95% CI = [0.26, 1.24], for Surprise; *p* < .001, 95% CI = [0.78, 1.76], for Neutral-Peacefulness), Australian female participants (*p* = .002, 95% CI = [0.28, 1.21], for Happiness; *p* = .008, 95% CI = [0.16, 1.10], for Surprise), and Chinese female participants (*p* = .005, 95% CI = [0.19, 1.07], for Fear; *p* < .001, 95% CI = [0.33, 1.21], for Neutral-Peacefulness).

*Three-way interaction.* One^[[11]](#footnote-11)^ three-way interaction effect was significant but related only to the stimulus traits.

*Two-way interaction.* Two^[[12]](#footnote-12)^ two-way interactions were identified, which were both qualified by the above higher-order interactions.

*Main effect*. Three^[[13]](#footnote-13)^ main effects were significant, but their result interpretations were all qualified by the above interactions.

***Cognitive empathy***. *Three-way interaction.* Two^[[14]](#footnote-14)^ significant three-way interactions were observed. (1) Participant Culture × Stimulus Sex × Stimulus Emotion^1^ [*F* (5, 2874.03) = 4.14, *p* < .001]. The following effects were significant according to the pairwise comparisons. First, the Participant Culture effect was significant on both Male Anger (i.e., Australian > Chinese participant, *p* = .001, 95% CI = [0.25, 1.05]) and Male Surprise (i.e., Chinese > Australian participant, *p* = .006, 95% CI = [0.17, 0.97]). Second, the Stimulus Sex effect (i.e., all were female > male stimuli, all *p*s < .001) was observed with both Australian participants (95% CI = [1.26, 1.91], for Surprise; 95% CI = [1.26, 1.91], for Neutral-Peacefulness) and Chinese participants (95% CI = [0.51, 1.14], for Surprise; 95% CI = [0.94, 1.55], for Neutral-Peacefulness).

*Two-way interaction.* Four^[[15]](#footnote-15)^ significant two-way interactions were identified. (1) Participant Sex × Stimulus Emotion^1^ [*F* (5, 2874.01) = 2.80, *p* = .016]. According to the pairwise comparisons, the Participant Sex effect (i.e., female > male participant) was significant for four emotions, namely, Happiness (*p* < .001, 95% CI = [0.27, 0.94]), Anger (*p* = .006, 95% CI = [0.14, 0.80]), Surprise (*p* < .001, 95% CI = [0.27, 0.93]), and Neutral-Peacefulness (*p* = .003, 95% CI = [0.17, 0.83]).

*Main effect*. There were four^[[16]](#footnote-16)^ significant main effects, and all were qualified by the above interaction effects.

***Perspective taking***. *Three-way interaction.* Two significant three-way interaction effects were found. (1) Participant Sex × Stimulus Sex × Stimulus Emotion^1^ [*F* (5, 2874.00) = 3.57, *p* = .003]. The pairwise comparisons revealed the following results. First, the Participant Sex effect was significant on both Female Happiness (i.e., female > male participant, *p* < .001, 95% CI = [0.81, 2.04]) and Female Surprise (i.e., female > male participant, *p* = .009, 95% CI = [0.20, 1.43]). Second, with both male and female participants, the Stimulus Sex effect (i.e., all were female > male stimuli) was significant on both Surprise (i.e., *p* = .006, 95% CI = [0.19, 1.14], for male participant; *p* < .001, 95% CI = [0.64, 1.54], for female participant) and Neutral (i.e., both female > male stimuli, and both *p*s < .001; 95% CI = [1.10, 2.04], for male participant; 95% CI = [0.83, 1.72], for female participant). In addition, male participants showed a sex in-group favor on Happiness (i.e., male > female stimuli, *p* < .001, 95% CI = [0.42, 1.36]). Meanwhile, female participants had a sex out-group favor on Sadness (i.e., male > female stimuli, *p* = .002, 95% CI = [0.24, 1.13]).

(2) Participant Culture × Participant Sex × Stimulus Emotion^1^ [*F* (5, 2874.01) = 2.60, *p* = .024]. Pairwise comparisons for this marginal significant interaction revealed the following results. First, the Participant Culture effect on Fear was significant with both male participants (i.e., Chinese male > Australian male participants, *p* = .010, 95% CI = [0.25, 1.76]) and female participants (i.e., Chinese female > Australian female participants, *p* < .001, 95% CI = [1.06, 2.49]). Second, the Participant Sex effect on Happiness was significant with Australian participants (i.e., Australian female > Australian male participant, *p* = .007, 95% CI = [0.28, 1.79]).

*Two-way interaction.* Three^[[17]](#footnote-17)^ significant two-way effects were identified. Nevertheless, these two-way interactions were either qualified by the above three-way interactions or related only to stimulus effects.

*Main effect.* Two^[[18]](#footnote-18)^ main effects were significant, with both being qualified by the above interactions.

1. Pairwise comparisons for all Stimulus Emotion effects and one subsequent Empathy Task effect were too long to be presented in this document (see Supplementary Document 5 for these pairwise comparisons). [↑](#footnote-ref-1)
2. The other three interactions were (4) Participant Culture × Stimuli Emotion [*F* (5, 5874.00) = 22.22, *p* < .001], (5) Stimulus Ethnicity × Stimulus Emotion [*F* (5, 5874.24) = 5.83, *p* < .001], and (6) Empathy Task × Stimulus Emotion [*F* (5, 5873.99) = 6.05, *p* < .001]. The interactions of (4) and (5) were qualified by a three-way interaction reported in the main text and were not examined further. The interaction of (6) did not relate to participant traits (see Supplementary Document 5 for its pairwise comparison results). [↑](#footnote-ref-2)
3. The two main effects were (1) Stimulus Sex [*F* (1, 5874.61) = 12.46, *p* < .001] and (2) Stimulus Emotion [*F* (5, 5875.26) = 128.68, *p* < .001] (see Supplementary Document 5 for details). [↑](#footnote-ref-3)
4. The third interaction, (3) Stimulus Ethnicity × Stimulus Emotion [*F* (5, 2874.09) = 4.00, *p* = .001], did not relate to participant traits (see Supplementary Document 5 for its pairwise comparison results). [↑](#footnote-ref-4)
5. The other main effect was (2) Stimulus Emotion [*F* (5, 2874.48) = 68.79, *p* < .001] (its result interpretations were qualified by the two-way interactions as per the main text and the previous footnote). [↑](#footnote-ref-5)
6. The other two interactions were (2) Participant Culture × Stimulus Sex [*F* (1, 2873.99) = 5.35, *p* = .021] (its result interpretations were qualified by the four-way interaction as per the main text) and (3) Stimulus Ethnicity × Stimulus Emotion [*F* (5, 2874.15) = 3.57, *p* = .003] (it is not related to participant traits, see Supplementary Document 5 for its pairwise comparisons). [↑](#footnote-ref-6)
7. The two main effects were (1) Stimulus Sex [*F* (1, 2874.41) = 7.40, *p* = .007] and (2) Stimulus Emotion [*F* (5, 2874.85) = 101.17, *p* < .001]. [↑](#footnote-ref-7)
8. The other four interactions were (3) Participant Culture × Stimulus Sex × Stimulus Emotion [*F* (5, 8874.04) = 5.03, *p* < .001], (4) Participant Sex × Stimulus Sex × Stimulus Emotion [*F* (5, 8874.02) = 4.25, *p* < .001], (5) Empathy Task × Stimulus Sex × Stimulus Emotion [*F* (10, 8874.00) = 3.33, *p* < .001], and (6) Stimulus Ethnicity × Stimulus Sex × Stimulus Emotion [*F* (5, 8874.10) = 11.70, *p* < .001]. In terms of interactions (3) and (4), both were qualified by the four-way interaction as per the main text. In terms of interactions (5) and (6), both were not related to participant traits (see Supplementary Document 5 for their pairwise comparison results). [↑](#footnote-ref-8)
9. The seven interactions were (1) Empathy Task × Participant Culture [*F* (2, 8874.00) = 12.61, *p* < .001], (2) Participant Culture × Stimulus Emotion [*F* (5, 8874.53) = 8.14, *p* < .001], (3) Participant Sex × Stimulus Emotion [*F* (5, 8874.02) = 3.34, *p* = .005], (4) Empathy Task × Stimulus Sex [*F* (2, 8874.00) = 5.56, *p* = .004], (5) Empathy Task × Stimulus Emotion [*F* (10, 8874.00) = 40.78, *p* < .001], (6) Stimulus Ethnicity × Stimulus Emotion [*F* (5, 8874.34) = 7.08, *p* < .001], and (7) Stimulus Sex × Stimulus Emotion [*F* (5, 8874.51) = 45.34, *p* < .001]. [↑](#footnote-ref-9)
10. The five main effects were (1) Empathy Task [*F* (2, 8874.00) = 341.96, *p* < .001], (2) Participant Sex [*F* (1, 125.01) = 9.72, *p* = .002], (3) Stimulus Ethnicity [*F* (1, 8874.23) = 16.03, *p* < .001], (4) Stimulus Sex [*F* (1, 8876.38) = 74.78, *p* < .001]), and (5) Stimulus Emotion [*F* (5, 8875.97) = 39.17, *p* < .001]. [↑](#footnote-ref-10)
11. The three-way interaction was Stimulus Ethnicity × Stimulus Sex × Stimulus Emotion [*F* (5, 2874.03) = 6.15, *p* < .001] (see Supplementary Document 5 for its pairwise comparisons). [↑](#footnote-ref-11)
12. The two interactions were (1) Participant Culture × Stimulus Emotion [*F* (5, 2874.13) = 3.30, *p* = .006] and (2) Stimulus Sex × Stimulus Emotion [*F* (5, 2874.13) = 8.31, *p* < .001]. [↑](#footnote-ref-12)
13. The three main effects were (1) Stimulus Ethnicity [*F* (1, 2874.06) = 12.44, *p* < .001], (2) Stimulus Sex [*F* (1, 2874.56) = 19.28, *p* < .001], and (3) Stimulus Emotion [*F* (5, 2874.47) = 71.22, *p* < .001]. [↑](#footnote-ref-13)
14. The other interaction, (2) Stimulus Ethnicity × Stimulus Sex × Stimulus Emotion [*F* (5, 2874.07) = 9.20, *p* < .001], did not relate to participant traits (see Supplementary Document 5 for its pairwise comparisons). [↑](#footnote-ref-14)
15. The other three interactions were (2) Participant Culture × Stimulus Emotion [*F* (5, 2874.38) = 6.12, *p* < .001], (3) Stimulus Ethnicity × Stimulus Emotion [*F* (5, 2874.25) = 6.40, *p* < .001], and (4) Stimulus Sex × Stimulus Emotion [*F* (5, 2874.38) = 33.54, *p* < .001]; their result interpretations were all qualified by the higher-order interactions as per the main text and the previous footnote. [↑](#footnote-ref-15)
16. The four main effects were (1) Participant Sex [*F* (1, 125.01) = 10.24, *p* = .002], (2) Stimulus Ethnicity [*F* (1, 2874.16) = 8.96, *p* = .003], (3) Stimulus Sex [*F* (1, 2875.74) = 102.05, *p* < .001], and (4) Stimulus Emotion [*F* (5, 2875.45) = 81.97, *p* < .001]. [↑](#footnote-ref-16)
17. The three two-way interactions were (1) Participant Culture × Stimulus Emotion [*F* (5, 2874.29) = 9.97, *p* < .001], (2) Stimulus Sex × Stimulus Emotion [*F* (5, 2874.29) = 22.46, *p* < .001], and (3) Stimulus Ethnicity × Stimulus Emotion [*F* (5, 2874.18) = 4.03, *p* = .001]. In terms of (1) and (2), their interpretations were both qualified by the higher-order interactions as per the main text. In terms of (3), it did not relate to participant traits (see Supplementary 4 for pairwise comparisons). [↑](#footnote-ref-17)
18. The two main effects were (1) Stimulus Sex [*F* (1, 2875.35) = 11.83, *p* < .001] and (2) Stimulus Emotion [*F* (5, 2875.12) = 21.35, *p* < .001]. [↑](#footnote-ref-18)
